# Exploring the Efficacy and Potential Mechanisms of Topical *Periplaneta americana* (L.) Extract in Treating Androgenetic Alopecia in a Mouse Model: A Systems Pharmacology and Skin Microbiome Analysis

**DOI:** 10.3390/biology14070831

**Published:** 2025-07-08

**Authors:** Tangfei Guan, Xin Yang, Canhui Hong, Peiyun Xiao, Yongshou Yang, Chenggui Zhang, Zhengchun He

**Affiliations:** 1 Yunnan Provincial Key Laboratory of Entomological Biopharmaceutical R&D, College of Pharmacy, Dali University, Dali 671000, China; 2 National-Local Joint Engineering Research Center of Entomoceutics, Dali 671000, China; 3 West China School of Public Health, Sichuan University, Chengdu 610041, China; 4 West China Fourth Hospital, Sichuan University, Chengdu 610041, China

**Keywords:** *Periplaneta americana* (L.), androgenic alopecia, network pharmacology, transcriptomics, non-target metabolomics, microbiota ecological balance

## Abstract

Androgenetic alopecia (AGA), the most prevalent form of hair loss worldwide, faces significant therapeutic challenges due to high costs and limited efficacy of current interventions, necessitating safer and more effective solutions. *Periplaneta americana* (L.)-derived PA-011, endowed with anti-inflammatory and antioxidant properties, has demonstrated notable hair growth-promoting effects in AGA mouse models. This study employed LC-MS/MS, peptidomics, and network pharmacology to characterize PA-011’s chemical composition and predict its potential targets in AGA pathogenesis. Using Western blot and RT-qPCR, PA-011 intervention significantly inhibited inflammatory responses and oxidative stress levels in mouse skin tissues. Concurrently, PA-011 activated the proliferative potential of hair follicle stem cells, as demonstrated by upregulated expression of the cell proliferation marker Ki67, and activated the Wnt/β-catenin signaling pathway in DHT-induced AGA mice. Transcriptomic and metabolomic analyses revealed multi-target effects of PA-011, including modulation of PI3K-Akt/MAPK pathways, pentose phosphate metabolism, and amino acid biosynthesis. 16S rRNA sequencing and metagenomic analysis showed that AGA disrupts skin microbial homeostasis, while PA-011 intervention normalized the microbiota composition. Topical application of PA-011 promoted robust hair regrowth without detectable toxicity in safety assessments. This preclinical study establishes PA-011 as a promising candidate for AGA therapy, warranting further translational investigation.

## Simple Summary

Androgenetic alopecia (AGA), the most prevalent form of hair loss, faces challenges such as high costs and limited efficacy in current treatments. This study demonstrates that PA-011, a derivative of *Periplaneta americana* (L.), exhibits promising therapeutic potential in AGA mouse models. Using a DHT-induced AGA mouse model combined with multi-omics approaches (LC-MS/MS, transcriptomics, metabolomics) and 16S rRNA microbiome analysis, we found that PA-011 promotes hair follicle stem cell proliferation (via upregulated Ki67 expression) by activating the Wnt/β-catenin pathway while suppressing oxidative stress and inflammation. Mechanistic studies revealed its ability to regulate the PI3K-Akt/MAPK signaling pathways, improve pentose phosphate metabolism and amino acid biosynthesis, and maintain skin microbial homeostasis. Safety assessments confirmed no toxicity with topical application. This preclinical study highlights PA-011 as a multi-target therapeutic candidate for AGA, offering a novel strategy for future treatment development.

## 1. Introduction

Androgenetic alopecia (AGA) is the most common hair loss disorder globally; it can severely affect patient quality of life regardless of race or gender. It is mainly caused by complex factors, such as genetics, hormonal imbalance, immunity, and inflammation [1], and it can have negative effects ranging from anxiety, depression, and trauma to self-esteem [2]. There are several medical, surgical, light-based, and nutritional treatment options for slowing or reversing AGA progression; however, the relatively high cost of AGA treatment and its limited modalities and efficacy do not meet the needs and expectations of a wide range of patients.

The actual AGA pathogenesis is currently unknown; however, androgen-mediated apoptosis of the hair follicle is thought to be the main cause of AGA. In the hair follicle, the common androgen testosterone is metabolized by type II 5α-reductase to dihydrotestosterone (DHT), which has a higher affinity for the androgen receptor (AR), inducing dermal papilla cells (DPCs) to secrete interleukin-1α (IL-1α), tumor necrosis factor-α (TNF-α), and transforming growth factor-β1 protein (TGF-β1), inducing early termination of the growth phase [3] and ultimately leading to hair loss. Therefore, attenuating the inflammatory response, inhibiting androgenic testosterone production, and promoting growth factor expression are key strategies for AGA treatment.

The insect *Periplaneta americana* (L.) (PA) is a traditional Chinese medicinal insect first recorded in the ancient Chinese pharmacological text, *Shennong Ben Cao Jing* [4]. As documented in *Lu Chuan Ben Cao* [5], PA has been used for the elimination of stagnant blood, evacuation of hematomas, detoxification, and stimulation of urination and edema [6]. Its efficacy has been demonstrated in the treatment of ulcers, burn wounds, tuberculosis, ulcerative colitis, cardiovascular disease, impaired healing of cancer [6,7], dermatological conditions, and other ailments [8].

Modern pharmacological studies have demonstrated that American cockroach extract exerts a range of pharmacological effects, including anti-inflammatory, antimicrobial, and antioxidant properties, as well as activation of EGF and vascular endothelial growth factor (VEGF) secretion in vivo and ex vivo, acceleration of wound healing, collagen synthesis, and angiogenesis [9,10,11]. To some extent, these effects are aligned with the therapeutic goals of AGA. Consequently, the hair growth-promoting activity of the *P. americana* (L.) extract (PAE) was screened to obtain PA-011. To further investigate the role and mechanism of hair growth promotion in AGA mice, we first analyzed the chemical composition of PA-011 using LC-MS/MS assay and peptidomics testing. We then predicted the interactions between the PA-011 components and AGA targets and their corresponding pathways through network pharmacology. The AGA model was prepared by subcutaneous injection of DHT into the backs of the C57BL/6J mice. Following the administration of PA-011 to depilated areas of the mice, hair growth was observed in each group. The levels of oxidative stress indicators and inflammatory factors in the skin tissues of the depilated areas were determined using an ELISA kit. The status and number of hair follicles were observed by hematoxylin and eosin (HE) staining of skin tissue sections. Furthermore, immunofluorescence staining was used to observe the proliferation and apoptosis of hair follicle cells, evaluating the effectiveness of PA-011 in AGA. Moreover, the activation of the Wnt/β-catenin pathway was validated by Western blot and RT-qPCR. Transcriptomic and metabolomic analyses were performed on skin tissues, and the mechanism of PA-011 in AGA was investigated by combining microbiota sequencing. Finally, comprehensive safety assessments of topical PA-011 were conducted via acute dermal toxicity tests in mice as well as skin irritation tests in guinea pigs and rabbits.

## 2. Materials and Methods

### 2.1. Materials

Dihydrotestosterone (DHT) was purchased from Beijing Solepol Technology Co., Ltd. (Beijing, China). Minoxidil was purchased from Beijing Nokai Technology Co., Ltd. (Beijing, China). 2-chloro-L-phenylalanine was purchased from Aladdin Reagent Co., Ltd. (Shanghai, China). Isoflurane was purchased from Reward Technology Co., Ltd. (Jinan, China). Vascular endothelial growth factor (VEGF), Hepatocyte Growth Factor (HGF), alkaline phosphatase (ALP/AKP), estradiol (E2), malondialdehyde (MDA), tumor necrosis factor (TNF-α), interleukin 6 (IL-6), total superoxide dismutase (SOD), and glutathione peroxidase (GSH-PX) were purchased from Nanjing Jianjian Institute of Bioengineering (Nanjing, China). Primary antibody Ki67, Terminal Deoxynucleotidyl Transferase dUTP End Labeling (TUNEL) kit, and DAPI staining were purchased from Wuhan Sevier Bio-technology Co., Ltd. (Wuhan, China). RIPA lysis buffer (Beyotime Biotechnology, P0013B, Shanghai, China), phosphatase inhibitors I and II (MedChemExpress, HY-K0021, HY-K0022, Monmouth Junction, NJ, USA), SDS-PAGE protein loading buffer (Beyotime, P0015L, Shanghai, China), and 10% PAGE gel rapid preparation kit (Apexbio Biotechnology, PG212, Shanghai, China) were purchased from technology companies. The primary antibodies used included β-actin (mouse-derived, GB12001), Wnt7a (rabbit-derived, A14194), and Wnt3a (rabbit-derived, A0642) (Abclonal, Wuhan, China). Primers for genes *GAPDH*, *Akt*, *Wnt7a*, and *β-catenin* were synthesized by Shanghai Servicebio Technology (Wuhan, China). Minoxidil (Icnbio Technology, AO447, Beijing, China), isoflurane (Ante Animal Husbandry Co., Ltd., 20230905, Jinan, China), and 1-chloro-2,4-dinitrobenzene (Titan Scientific, 91824A, Shanghai, China) were also used. Olive oil was purchased from Yihai Jiali Food Marketing Liability Company (Guangzhou, China). Methanol and acetonitrile were both high-performance liquid chromatography-grade and purchased from Thermo Fisher Scientific (Waltham, MA, USA). Ultrapure water was prepared using a Milli-Q system (Massachusetts, MA, USA).

### 2.2. Preparation of PA-011

The PA body was purchased from Yunnan Jingxin Biotechnology Co., Ltd. (Dali, China). The dried PA bodies were crushed and extracted with 80% ethanol three times the mass. The ethanol solution obtained from the combined extraction was concentrated under reduced pressure and 60 °C to obtain the PA extract. After defatting the extract, ethanol six times the mass of the extract was adjusted to a solution with a final alcohol concentration of 45%, which was left to rest overnight at 4 °C. Afterward, the insoluble matter was filtered out, and the solution was concentrated at 60 °C under reduced pressure to obtain freeze-dried PA-011 extract.

### 2.3. Identification of the Chemical Composition of PA-011

Small- and medium-sized molecular compounds were detected in PA-011. The samples were dissolved using a methanol solution containing 2-chloro-L-phenylalanine that was mixed, ground, sonicated using a tissue grinder, and centrifuged at a low temperature. The supernatant was filtered to obtain the samples.

The samples were then analyzed using the Vanquish ultra-high-performance liquid phase system. The flow rate, column temperature, and sample size were set as per a previous study [12]. In the positive ion mode, the mobile phase was 0.1% acetonitrile formate (B2) and 0.1% water formate (A2). In the negative ion mode, the mobile phase was acetonitrile (B3) and 5 mM ammonium formate water (A3) [13], with a gradient elution as presented in Table 1 [14]. Using a Thermo Q Exactive mass spectrometry detector with ESI, positive and negative ion modes were used to collect data, as previously described [15].

For the detection of polypeptide components in PA-011, the mobile phase consisted of A = 100% water + 0.1% formic acid and B = 80% acetonitrile + 0.1% formic acid [16]. Elution conditions are shown in Table 2. The QExactive HF-X mass spectrometer and Nanospray Flex™ (NSI) (Thermo Fisher Scientific, Waltham, MA, USA) ion source were used. Detection methods came from the literature [17]. Peptide sequences were obtained by de novo analysis.

### 2.4. Network Pharmacology Analysis and Molecular Docking Simulation

#### 2.4.1. Screening of Potentially Active Compounds and Compound Prediction

Small-molecule compounds from PA-011 (identified by LC-MS/MS) were screened for SMILES in PubChem (https://pubchem.ncbi.nlm.nih.gov/, accessed on 6 April 2024), entered into SwissADME (http://www.swissadme.ch/index.php, accessed on 8 April 2024), and filtered for therapeutic targets (probability > 0.01). Peptide sequences from the analysis (top 100 with −10lg*p* value and de novo, confidence > 95) were integrated, input into Emboss for protein information, and screened [18,19,20,21] for target information (probability > 0.01). SEA (https://sea.bkslab.org/, accessed on 9 April 2024) was used for peptide target prediction. Finally, integrated and de-weighted small-molecule and peptide target protein information was obtained.

#### 2.4.2. AGA Target Acquisition

Androgenic alopecia was selected as the search term and searched in the OMIM (https://omim.org/, accessed on 8 April 2024), GeneCards (www.genecards.org/, accessed on 8 April 2024), and DisGeNET databases (https://www.disgenet.org/search, accessed on 8 April 2024). The results of the three databases were incorporated, and repetitive disease target information was removed to obtain disease gene results. Disease targets and peptide prediction targets were imported into the Venny 2.1.0 tool (https://bioinfogp.cnb.csic.es/tools/venny/, accessed on 8 April 2024) to obtain the intersection gene information of each peptide on AGA disease, and the obtained disease targets and active ingredient targets were screened to obtain the targets of interaction between active ingredients and the disease.

#### 2.4.3. Ingredient-Pharmacological-Target-Disease Pathway Network Construction

The obtained small molecule compounds, their target info, peptides, peptide target info, disease intersection targets, and KEGG pathway target info were used to build a component-pharmacodynamic-target-disease pathway network worksheet and integrate it into a tape file. The data was imported into Cytoscape 3.9.1 to construct a network diagram. Additionally, CentiScaPe 2.2 (https://apps.cytoscape.org/apps/centiscape, accessed on 10 April 2024), a Cytoscape 3.9.1 plugin (https://cytoscape.org/download.html, accessed on 11 April 2024), was used to calculate relationships among small molecule compounds, peptides, pharmacodynamic targets, and disease pathways.

#### 2.4.4. Protein Interaction (PPI) Network Construction and Analysis

The intersecting disease targets were entered into the protein interaction search and prediction database STRING (https://string-db.org/, accessed on 11 April 2024). The unconnected nodes in the network were hidden, and the remaining parameters were set at default settings. The relevant data were exported. The protein network was analyzed using Centiscape 2.2 in Cytoscape 3.9.1, using the plug-in default filtering conditions: Degree (>14.432), Closeness (>0.003678), and Betweenness (>154.224).

#### 2.4.5. Gene Ontology (GO) and Kyoto Encyclopedia of Genes and Genomes (KEGG) Pathway Enrichment Analyses

Using DAVID (https://David.ncifcrf.gov/, accessed on 12 April 2024), intersecting disease target information was imported into the database, OFFICIAL_GENE_SYMBOL was selected from DAVID, and GO analysis and KEGG pathway enrichment analysis were conducted. We used the online biological information platform microscopic letter enrichment results for visualization processing (http://www.bioinformatics.com.cn/, accessed on 12 April 2024).

#### 2.4.6. Molecular Docking

To verify the binding activity between the core target protein and the corresponding active ingredient, AutoDock Vina was used for molecular docking. The structure file of the target protein and active ingredient was obtained from the PDB database (https://www.rcsb.org/, accessed on 13 April 2024), and then the protein was dehydrolyzed and deligand treated with PyMoL 4.6.0 software (https://www.pymol.org/, accessed on 12 April 2024). The active site was selected with the original ligand of the target protein as the center for molecular docking, and a heatmap was constructed. Finally, the results were visualized using PyMoL software.

### 2.5. Animal Experiments

#### 2.5.1. Experimental Animals and AGA Models

Sixty-five male and female C57BL/6J mice (purchased from Spife Biotechnology Co., Ltd., Beijing, China) weighing 18–22 g were selected. All animal studies were conducted in accordance with the Guidelines for the Care and Use of Laboratory Animals issued by the National Institute of Health, and the protocol was approved by the Laboratory Animal Ethics Committee, Dali University (Dali, China) (Approval No. 2024-PZ-003). Animals were raised under specific pathogen-free conditions at 22–25 °C and a 12-h light-dark cycle, with plenty of water, and food provided ad libitum.

After 7 d of adaptive feeding, female and male mice were divided randomly into ten groups: male and female blank group, model group, positive control group (male 5% minoxidil, female 2% minoxidil), PA-011 low-dose group (1%, PA-011L), and PA-011 high-dose group (4%, PA-011H), with 13 animals in each group.

Eight groups of AGA mice (excluding two blank groups) received subcutaneous DHT injections (1 mg/animal/2 d, in olive oil), whereas the blank group mice received an equal volume of olive oil [22,23]. After 5 d, all mice were anesthetized. Rosin and paraffin (1:1 mass ratio) were melted and used to coat an area of 3 cm × 4 cm on the backs of mice. After solidification, the hair was removed. DHT/oil injections continued post-hair removal until day 21. On the 2nd day of hair removal, a matrix solution (water/anhydrous ethanol/1,2-propylene glycol = 3:5:2) was applied to the shaved area of blank/model male/female mice. According to the literature, the positive female group received 2% minoxidil, and the positive male group received 5% minoxidil [24]. The male/female PA-011L group received 1% PA-011 solution, and the PA-011H group received 4% PA-011 solution, all administered twice daily until the end. On day 21, to compare the efficacy of PA-011, hair score was used to determine that the hair growth cycle had entered the telogen phase. Mice were then sacrificed, and skin tissues from depilated sites and visceral tissues were collected for subsequent analysis. Mice were anesthetized with isoflurane throughout (for hair removal, photography, and euthanasia).

#### 2.5.2. Epigenetic Evaluation of PA-011 on Hair Growth in AGA Mice

**Figure 1:**
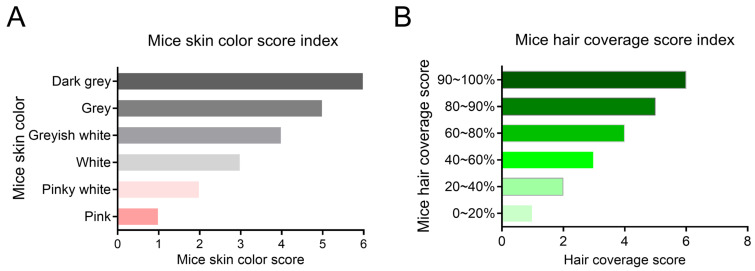
Apparent evaluation of hair growth in mice: (**A**) skin color scoring criteria; (**B**) hair coverage rating criteria.

On days 3, 8, and 13 after local administration of the drug following hair removal, the hair loss area on the back of the mice was photographed after anesthesia, and the hair growth status of the mice was recorded. The hair coverage area and skin color of mice in each group were scored starting from hair removal, and the scoring criteria are shown below (Figure 1).

#### 2.5.3. Determination of Mouse Biochemical Indexes

On day 21 of administration, euthanasia of mice followed institutional animal ethics guidelines using anesthetics. Blood and target skin tissues were collected with sterile instruments, with hair/fat removed. Tissues were frozen in liquid nitrogen and stored at −80 °C. Frozen tissues were washed in pre-chilled PBS, transferred to homogenizers, and grinding beads and an appropriate RIPA buffer containing protease and phosphatase inhibitors were added. Homogenization occurred at ~20,000 rpm for 10–15 cycles (5–10 s/cycle, 30-s ice intervals). Samples were centrifuged at 12,000–14,000 rpm for 10–15 min at 4 °C, supernatants were collected, and protein concentration was determined by BCA assay. Supernatants were used for ELISA (VEGF, HGF, MDA, TNF-α, IL-6) and activity kits (SOD, GSH-PX, ALP). Blood was incubated at RT for 1 h, then centrifuged at 3500× *g* for 10 min at 4 °C to obtain serum, with subsequent assays following kit protocols.

#### 2.5.4. Skin Pathology Observation and Immunofluorescence Detection in Mice

The murine skin (days 15 and 21) was immersed in 4% paraformaldehyde for 24 h and paraffin-embedded. After wax-embedding, sectioning, dewaxing, and hydration, the skin sections were placed on glass slides. Then, they were stained with a hematoxylin and eosin (HE) staining solution, dehydrated, cleared, and sealed. Terminal and Cui-ui hair follicle numbers were counted via microscopy, and ratios were calculated.

Take the same wax block stained with HE, paraffin sections were deparaffinized and washed in xylene (30 min), anhydrous ethanol (15 min), and distilled water. The samples were placed in a citric acid repair solution, washed with PBS, shaken dry, incubated with a membrane-breaking solution (20 min, RT), placed in PBS (pH 7.4), and washed thrice on a shaker (5 min each). After incubation, cells were stained as per the TUNEL kit instructions and observed under a fluorescence microscope to capture images (DAPI-stained nuclei: blue under UV, green under Tunel, red under Ki67).

##### 2.5.5. mRNA Expression Detection and Analysis by Real-Time Quantitative Polymerase Chain Reaction (RT-qPCR)

RNA was extracted using Trizol, and its concentration and purity were detected via NanoDrop 2000 (Thermo Fisher). Reverse transcription was performed as per the kit instructions (25 °C, 5 min; 42 °C, 30 min; 85 °C, 5 s). A 0.1-mL PCR plate was used with 7.5 μL 2 × SYBR Green qPCR Master Mix [None ROX], 1.5 μL 2.5 μM primers, 2.0 μL cDNA, and 4.0 μL nuclease-free water. The membranes were sealed post-sampling. Amplification was performed using fluorescence qPCR (Stage 1: 95 °C, 30 s pre-denature; Stage 2 [40 cycles]: 95 °C, 15 s denature, 60 °C, 30 s anneal/extend; Stage 3: 65 °C → 95 °C melting curve, 0.5 °C fluorescence collection). Expression signals were analyzed. The primer sequences are listed in Table 3.

#### 2.5.6. Western Blot (WB)

The total protein from skin tissue was extracted. Skin was washed 2–3 times with cold PBS, homogenized in 10× lysate, iced 30 min, and centrifuged (12,000 rpm, 4 °C, 10 min). Supernatant was used as the protein solution, mixed with 5× loading buffer (4:1), denatured in boiling water for 15 min, and stored at −20 °C. Proteins were separated by SDS-PAGE and transferred to a PVDF membrane in ice water. The membrane was incubated with 5% skim milk for 2 h, then with the primary antibody overnight and secondary antibody the next day, and cleaned with Tris buffer + Tween. Protein expression was detected by ECL and analyzed by ImageJ v6.0.

#### 2.5.7. Analysis of Skin Tissue Transcriptomic Test

Total RNA was extracted from skin using Trizol (Thermo Fisher Scientific, Invitrogen, MA, USA). Quality control and integrity testing were performed with NanoDrop ND-2000 (Thermo Fisher) and RNA-specific agarose electrophoresis using Agilent Bioanalyzer 2100 (New England Biolabs Inc.; Ipswich, MA, USA). Total RNA (1 µg) was selected for cDNA library construction with a NEBNext Ultra II RNA Library Prep Kit (Illumina). Library quality was tested using the Agilent 2100 Bioanalyzer and Agilent High-Sensitivity DNA Kit. The mixed library was diluted and quantified, and PE150 sequencing was performed on an Illumina NovaSeq 6000 by Shanghai Paisenol. After data files were obtained, topGO was used for GO enrichment analysis with hypergeometric distribution (*p* < 0.05) to determine significant GO terms and differential gene functions. KEGG enrichment analysis was performed using clusterProfiler v3.4.4 (https://bioconductor.org/packages/release/bioc/html/clusterProfiler.html, accessed on 10 June 2024) with a focus on pathways with *p* < 0.05.

#### 2.5.8. Non-Target Metabolomics Analysis of Skin Tissue

Sample extraction methods and detection conditions of LC-MS/MS are presented in Section 2.3. A follow-up test was conducted by Shanghai Peisenol Biotechnology Co., Ltd. (Shanghai, China) After data files were obtained, R package Ropls (v1.8.3) [25] was used for principal component analysis (PCA), partial least squares discriminant analysis (PLS-DA), and orthogonal partial least squares discriminant analysis (OPLS-DA) dimension reduction analysis of sample data. A score map was drawn to show metabolite composition differences among samples, and an overfitting test by substitution was performed. *p* values were calculated via statistical tests, VIP was calculated using OPLS-DA dimensionality reduction, and difference multiples were determined based on fold change. The influence and interpretation ability of metabolite components on sample classification and discrimination was measured to assist metabolite screening. When *p* < 0.05 and VIP > 1, metabolite molecules were considered significant. MetaboAnalyst (v3.0.8789) [26] was used for functional pathway enrichment and topological analysis of screened differential metabolites. The KEGG Mapper visualization tool was used to browse differential metabolites and pathway maps of enriched pathways.

#### 2.5.9. Joint Analysis of Skin Tissue Greening and Non-Target Metabolomics

The non-target metabolome and transcriptome of skin tissue were analyzed jointly. Firstly, correlation analysis was performed on the obtained non-target metabolome and transcriptome quantitative detection and analysis results, and then the differentially expressed metabolites and transcript information were extracted. Subsequently, the correlation results were screened and analyzed, and transcripts corresponding to related enzymes were extracted according to the metabolite information in the KEGG database, and the trends between the differentially expressed metabolites and transcripts were sorted. Furthermore, the common annotation pathways of different metabolites and genes in the two omics differential enrichment analyses were analyzed.

#### 2.5.10. Skin Microbiota Analysis

On the 21st day after hair removal, cotton swabs were wiped over the depilated skin. Genomic DNA was extracted using the CTAB method, and its purity and concentration were checked using 1% agarose gel electrophoresis. Qualified DNA samples were sent to Shanghai Piceno Biotechnology Co., Ltd. (Shanghai, China) for 16S rRNA and macrogene sequencing. Species and functional abundance tables facilitated the abundance cluster, PCoA, NMDS dimension reduction, and sample cluster analyses. Lefse biomarker and Dunn test analyses were conducted to investigate species and functional composition differences among samples [27].

### 2.6. Safety Evaluation of Topical Application of PA-011

#### 2.6.1. Experimental Animals

Twenty-four 8-week-old SPF-grade C57BL/6J mice (body weight 18–20 g), twenty-four clean-grade guinea pigs (250–350 g), and eight clean-grade New Zealand white rabbits (2.0–2.5 kg) were used. Half of each animal species were male and half were female. Additionally, twenty-four clean-grade fancy rats (200–300 g, equal numbers of males and females) were included. All animals were purchased from Hunan Taiping Biotechnology Co., Ltd. (Yiyang, China), with the experimental animal license number SCXK (Xiang) 2023-0011. Animals were housed and experiments were conducted at the Animal Experiment Center of Dali University under temperature/humidity-controlled conditions with a 12-h light/dark cycle. After a one-week adaptive feeding period, animals were randomly assigned to experimental groups. All experiments were approved by the Ethics Committee of Dali University, with approval numbers 2024-PZ-005 for skin irritation and sensitization tests and 2024-PZ-007 for acute toxicity tests.

#### 2.6.2. Acute Dermal Toxicity Test

We evaluated the acute dermal toxicity of PA-011 using the maximum concentrated dose method, in compliance with the Measures for the Approval of New Drugs and its Supplementary Provisions on Traditional Chinese Medicines. Twenty-four C57BL/6J mice (18–22 g, twelve males and twelve females) were randomly assigned to a vehicle control group and a PA-011 group (twelve mice per group). The hair on the central dorsum of each mouse was clipped and removed with depilatory cream, creating a 2.5 cm × 4 cm area for testing 24 h later. Mice in the PA-011 group received 0.2 mL of PA-011 (0.455 mL/g, 50-fold the daily clinical dose for a 70 kg adult) on the depilated skin, while control mice received the vehicle. Over 14 days, we monitored body weight, appearance, movement, mental state, appetite, feces, skin color, respiration, and secretions. After 14 days, we sacrificed the mice, collecting the treated skin tissue and major organs (heart, liver, spleen, lung, kidney) for histopathological analysis. The staining method is the same as the HE staining step in Section 2.5.4.

#### 2.6.3. Skin Sensitization Test

Twenty-four guinea pigs were randomized into three groups (n = eight/group): PA-011, damage control (1% dinitrochlorobenzene, DNCB), and vehicle control (blank group). Bilateral dorsal skin (5 cm × 2 cm) was depilated 24 h prior to treatment. Animals received 0.2 mL topical applications of test substances (PA-011/DNCB/vehicle) on the left depilated area, which were washed off after 6 h. Sensitization was repeated on days 7 and 14 using identical protocols. Fourteen days after the third sensitization, challenge testing was performed with 0.2 mL of 0.1% DNCB (damage control), PA-011, or vehicle. Skin reactions (erythema, edema, lesions) were scored daily for 3 days post-challenge. Skin tissues were harvested for HE staining as described in Section 2.5.4.

#### 2.6.4. Skin Irritation Test

Eight rabbits were randomly divided into two groups (four rabbits per group): a vehicle control (normal group) and a high dose (4%) (damage group). Twenty-four hours before the experiment, hair on both sides of the spinal column on the dorsum of all animals was removed with depilatory cream, creating an area of approximately 50 cm^2^, and a self-control design was adopted. In the intact skin group, no special treatment was applied after depilation, while in the damaged skin group, the area was disinfected with 75% ethanol, and a “#” shape was incised on both depilated sides using a size 16 sterile needle until slight bleeding occurred, ensuring consistent skin lesion severity on both sides. During the experiment, 4% PA-011 was evenly applied to the depilated area (the blank group received vehicle application), covered with non-irritating gauze, and fixed with adhesive tape. The treatment was reapplied once after a 6-h interval (twice in total) and administered continuously for 17 days. Observations and records included the presence of erythema and edema, pigmentation, petechiae, skin roughness, or thinning at the application site, along with their onset and resolution times. Histopathological examination of the skin was performed. The staining method was the same as the HE staining step in Section 2.5.4.

### 2.7. Statistical Analysis

The data are expressed as mean ± standard error of the mean (SEM) or in a bar chart in the ± SEM range. Statistical analysis and plotting were performed using GraphPad Prism 10 (GraphPad Software Inc., La Jolla, CA, USA). Student’s *t* test was used to compare differences between the two groups, and one-way analysis of variance was used to analyze differences between more than two groups. *p* < 0.05, indicated a significant difference, and comparison with the blank group was represented by *, where * *p* < 0.05, ** *p* < 0.01, *** *p* < 0.001, **** *p* < 0.0001. Compared with the model group, it was represented by #, where # *p* < 0.05, ## *p* < 0.01, ### *p* < 0.001, #### *p* < 0.0001.

## 3. Results

### 3.1. Composition of PA-011

LC-MS/MS analysis results showed that PA-011 contained 344 small molecule compounds, including 62 amino acids, polypeptides, and analogues; 38 organic acids and their derivatives; 22 fatty acids and conjugates; 22 nucleotides and their derivatives; 17 benzoic acids and their derivatives; 14 carbohydrates; 14 phenols; 13 alcohols; and 12 amino acids, in addition to 7 amines, 7 lipids, and other types of compounds (see Supplementary Materials for details). The total number of peptides identified in the peptitomic analysis was 16,515 (see Supplementary Materials for details).

### 3.2. Network Pharmacology Results

#### 3.2.1. Intersection Target Acquisition

According to the peptide screening criteria, 33 peptides were selected from the top 100 entries with the highest −10lg*p* values in the peptide database, and 102 peptides were filtered from 287 de novo-only peptides with a confidence score > 95%. After merging and removing duplicates, 129 unique peptide sequences were obtained.

**Figure 2:**
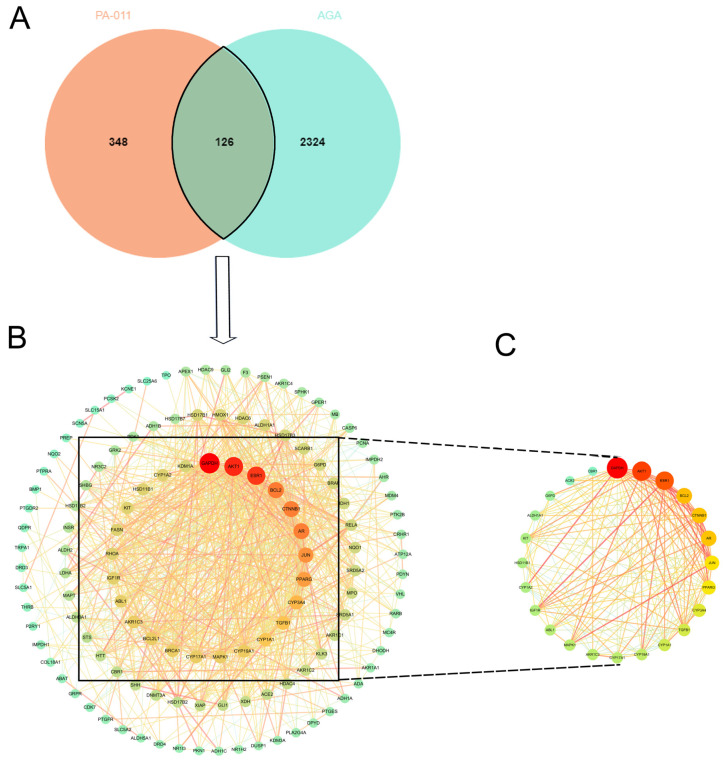
Screening of core targets. (**A**) Venn diagram showing intersection targets; (**B**) PPI network diagram; (**C**) screening core proteins. Both green and red represent targets. The darker the color and the larger the circle, the higher the degree value, and the more important the target.

A total of 474 drug targets were obtained by predicting the sequenced of PA-011 small molecule compounds and peptides, and a total of 2450 disease targets were obtained by integrating disease database information. In addition, 126 intersection targets were obtained by integrating Venn diagram analysis (Figure 2A). The obtained intersection targets were subjected to PPI network analysis, and a PPI network diagram with 125 nodes and 902 edges was obtained (Figure 2B). According to the default screening conditions of the Centiscape 2.2 software plug-in, the interaction network between 24 core protein nodes and 157 edges was screened (Figure 2C). Among them, MAPK1, Bcl-2, Akt, ESR1, and other core proteins that play a positive role in AGA therapy had strong correlations.

#### 3.2.2. Enrichment Analysis

**Figure 3:**
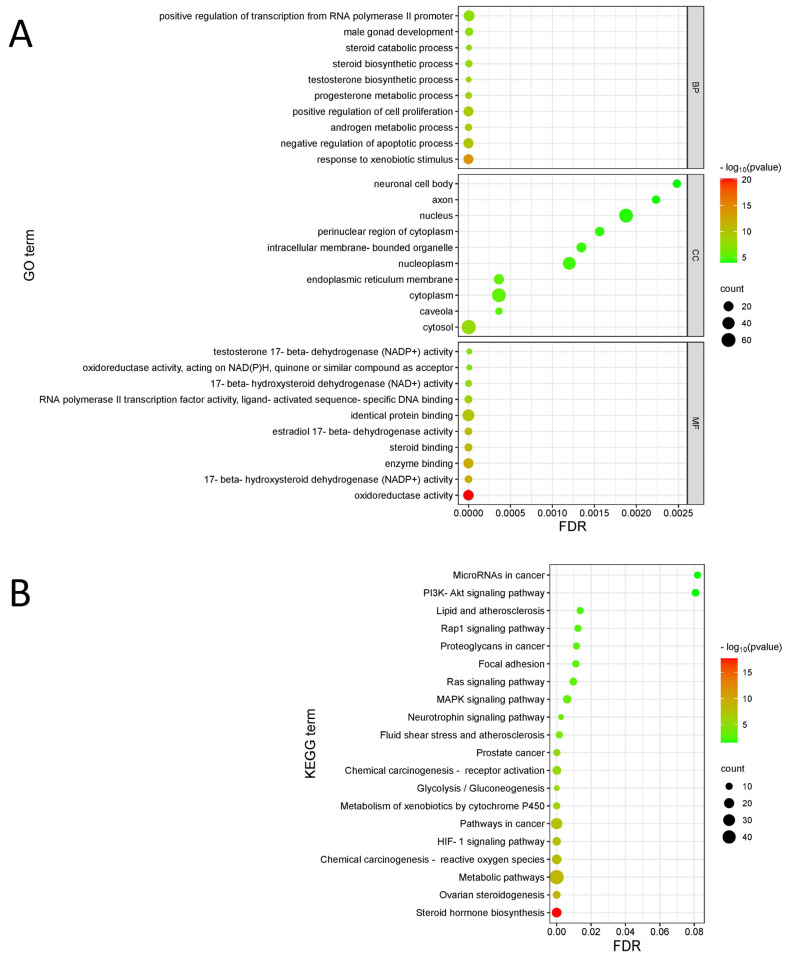
Enrichment analysis. (**A**) Bubble map of GO functional analysis; (**B**) KEGG channel enrichment bubble map.

Based on GO enrichment (Figure 3A) and KEGG enrichment analyses (Figure 3B), the biological process (BP) information from the GO enrichment analysis showed that PA-011 components were involved in negative regulation of apoptosis, active regulation of cell proliferation, male hormone metabolism, steroids and hormone effects in vivo, and anti-exogenous stimulation. In addition, PA-011 components had positive effects on AGA apoptosis, androgen inhibition, and anti-stress performance. In cell composition (CC) and molecular function (MF) information, PA-011 mainly acts on intercellular information transmission by participating in apoptosis, combines hormone signals, and balances AGA hormones, in turn generating cell protection and anti-stimulation effects. Finally, KEGG enrichment analysis and pathway information analysis showed that PA-011 could regulate the MAPK signaling pathway, P13K-Akt signaling pathway, and HIF-1 signaling pathway, which affect apoptosis; produce anti-inflammatory and anti-apoptosis effects; regulate cell metabolism; regulate steroid hormones; and provide other beneficial effects for AGA disease treatment.

#### 3.2.3. Component Target Path Correlation Network Analysis

**Figure 4:**
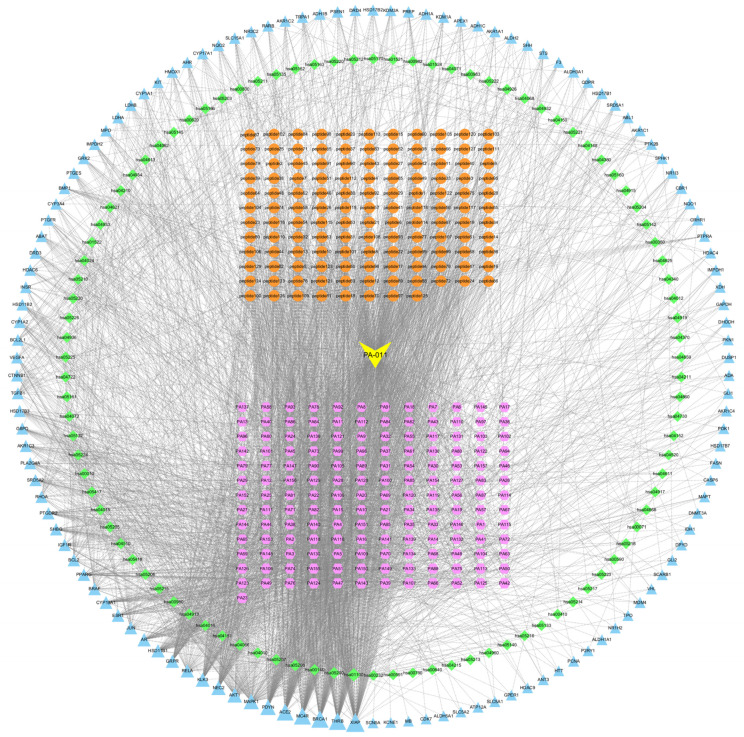
Drug-core-target-KEGG pathway network map.

The collected KEGG target information, intersection protein information, small molecule compounds, and polypeptide data are displayed in the network (Figure 4). PA-011 samples in yellow, disease targets in orange, small molecule compounds in blue, polypeptides in purple, and KEGG pathways in green are closely connected, indicating that PA-011 is highly correlated with AGA therapeutic targets.

#### 3.2.4. The Results of Molecular Docking

**Figure 5:**
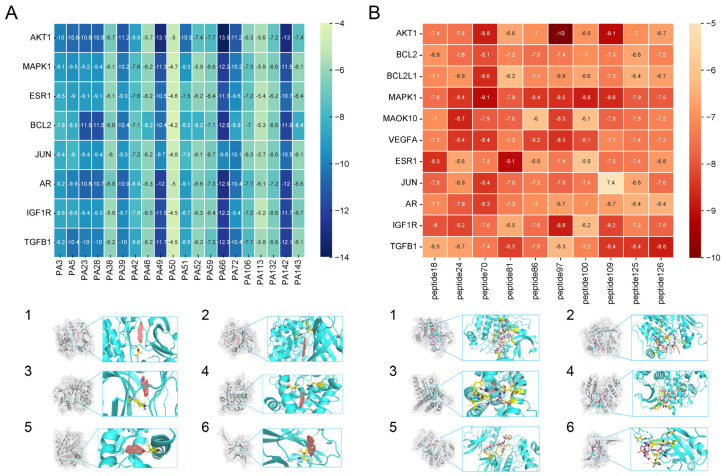
Molecular docking. (**A**) Small molecule compound docking fraction heat map; (**A1**) AKTI docked with sotalol; (**A2**) AKTI docked with 17a−estradiol; (**A3**) AKTI docked with dehydroepiandrosterone; (**A4**) AR docked with dehydroepiandrosterone; (**A5**) Bcl−2 docked with dehydroepiandrosterone; (**A6**) TGFB1 docked with dehydroepiandrosterone; (**B**) polypeptide molecular docking fraction heat map; (**B1**) AKT1 docked with peptide97; (**B2**) AKT1 docked with peptide109; (**B3**) ESR1 docked with peptide81; (**B4**) MAPK1 docked with peptide70; (**B5**) MAPK1 docked with peptide100; (**B6**) TGFB1 docked with peptide126.

According to the binding energy after molecular docking, the lower the binding energy between ligand and receptor, the more stable the binding and the stronger the binding activity. Six target proteins with higher degrees in PPI analysis and greater association with AGA were selected for docking with 20 small molecules (Table 4). In addition, six target proteins were selected according to the same conditions for molecular docking with the top 10 polypeptide sequences with a degree (Table 5). According to the docking results, the binding energy of 96.3% of the small molecule compounds and their targets was <−5 kcal/mol, and the binding energy of all polypeptides and their targets was <−5 kcal/mol. The visualization heat map of the docking results showed that the active ingredient PA-011 had strong binding activity with the AGA target. The small molecule compounds and peptides with binding energy < −9 kcal/mol were visualized using PyMoL software (Figure 5). Quantitative analysis data for key active compounds in PA-011 (such as 17α-estradiol, dehydroepiandrosterone, and 18 other compounds)—including LC-MS peak areas and concentration information in ppm units—have been used as Supplementary Material.

### 3.3. PA-011 Can Significantly Promote Hair Growth in AGA Mice

**Figure 6:**
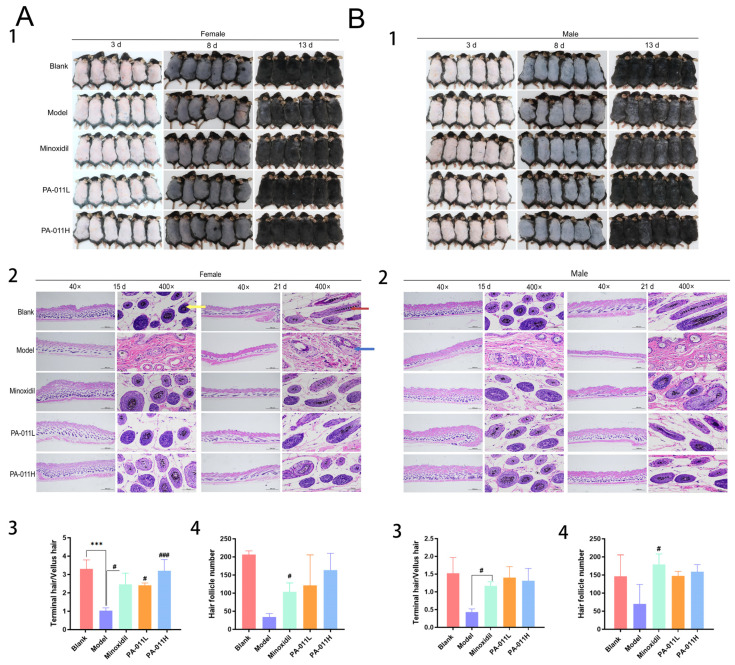
Hair growth status. (**A**) Hair growth status of female mice (n = 6); (**B**) hair growth status of male mice (n = 6); (1) hair growth status display (n = 6); (2) skin H&E staining (n = 3); (3) vellus hair ratio (n = 3); (4) total hair follicles (n = 3). 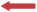 and 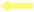 indicate terminal hair follicles; 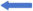 indicates vellus hair follicles. One-way analysis of variance (ANOVA) was performed. *** *p* < 0.001 vs. blank group; # *p* < 0.05, ### *p* < 0.001 vs. model group. Scale bars: 500 μm and 50 μm.

Mice in each group were photographed on day 3, day 8, and day 13 after hair removal (Figure 6). The results showed that compared with the blank group, the hair growth of mice in all groups injected with DHT was retarded to varying degrees; however, the hair growth rate of mice in all treatment groups was significantly higher than that in the model group. Among them, the hair growth status of the mice in the group treated with PA-011 was closer to that of the blank group.

The results of HE staining and hair follicle counting on the sacrificed mice on the 15th and 21st day after hair removal show that compared with the model group, total hair follicle number and final hair number of mice in the minoxidil group and PA-011 administration group were significantly higher, whereas vellus hair follicle number of mice in the minoxidil group and PA-011 administration group was not significantly different. However, hair follicles were larger, and the miniaturization and cavulation were reduced, with increased terminal/vellus hair ratio (Figure 6).

### 3.4. Hair Growth Coverage and Gross Weight Score

**Figure 7:**
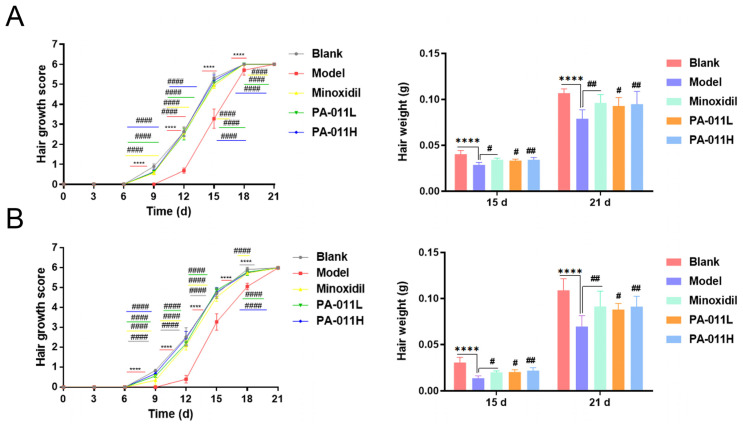
Hair growth coverage and gross weight score. (**A**) Hair coverage and gross weight scores of female mice (n = 5–8); (**B**) hair coverage and gross weight scores of male mice (n = 5–8). One-way analysis of variance (ANOVA) was performed. **** *p* < 0.0001 vs. blank group; # *p* < 0.05, #*# p* < 0.01, #### *p* < 0.0001 vs. model group.

Hair coverage of mice was scored every 3 d after hair removal (Figure 7). Compared with other groups, hair growth was slower and sparse in the model group. The hair in the same part of the hair shedding area of sacrificed mice in each group on the 15th and 21st days of hair removal was weighed, and the hair weight of mice in each drug administration group was greater than that in the model group (Figure 7).

### 3.5. PA-011 Can Increase E2 Content and ALP Activity in AGA Mice and Reduce the Level of Inflammatory Factors and Oxidative Stress in Skin

**Figure 8:**
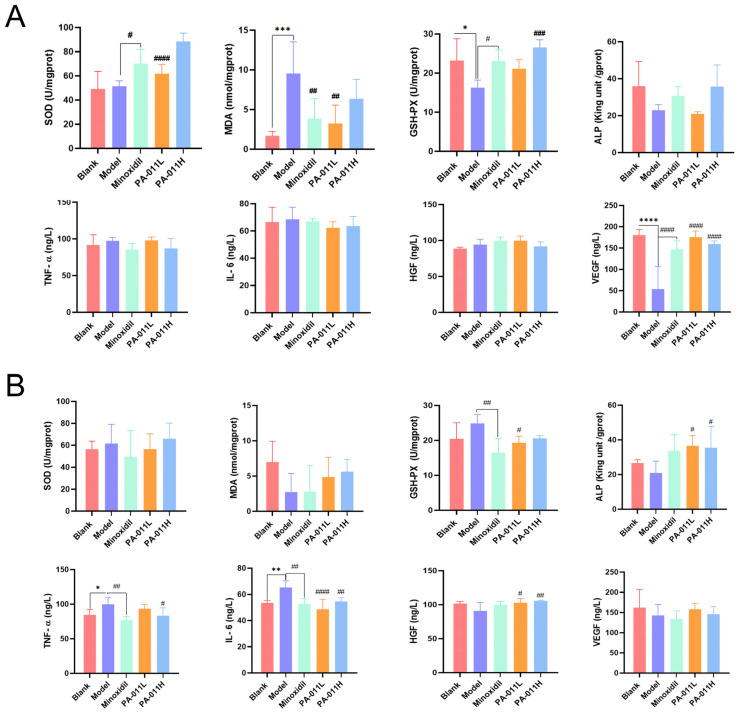
Detection of inflammation, oxidative stress, and active substances in serum and tissue of mice (n = 5). (**A**) Changes of factors in female mice; (**B**) changes of factors in male mice. One-way analysis of variance (ANOVA) was performed. * *p* < 0.05, ** *p* < 0.01, *** *p* < 0.001, **** *p* < 0.0001 vs. blank group; # *p* < 0.05, ## *p* < 0.01, ### *p* < 0.001, #### *p* < 0.0001 vs. model group.

The E2 content in serum; the levels of VEGF, HGF, MDA, TNF-α, and IL-6 in skin tissue SOD and GSH-PX activity; and dermal papilla activity marker (ALP) levels in hair follicles [28] were measured. Following PA-011 treatment, E2 content in serum and the expression levels of ALP and HGF in skin tissue increased significantly, whereas the expression of TNF-α and IL-6 decreased significantly. There was no significant change in serum E2 content of female mice (which may be related to the high E2 level in female mice); however, VEGF and HGF levels and SOD and GSH-PX activities in skin tissues were increased significantly, and MDA level was decreased significantly (Figure 8).

### 3.6. PA-011 Can Significantly Promote Proliferation and Decrease Apoptosis of Hair Follicle Cells

**Figure 9:**
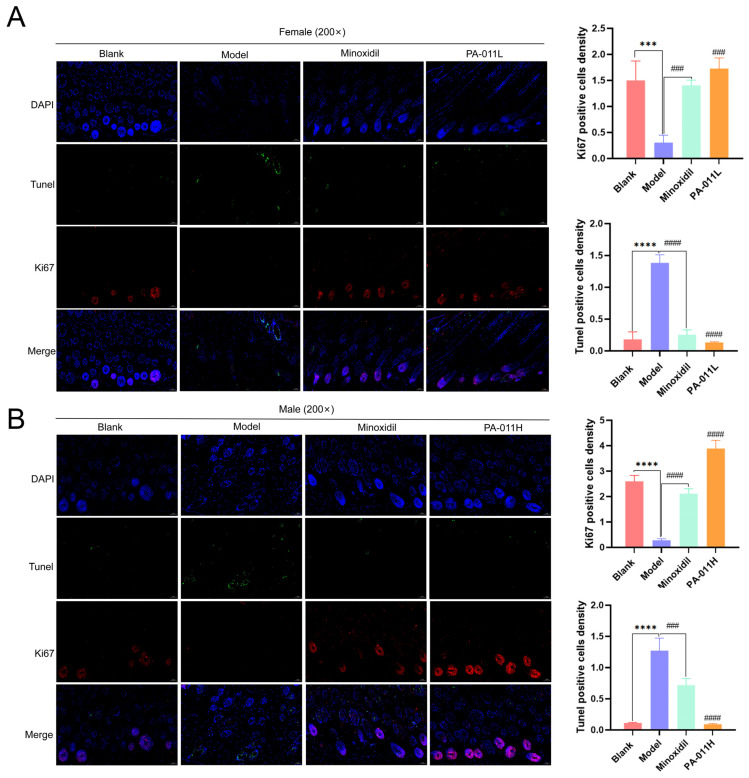
Skin Ki67 and TUNEL double immunofluorescence staining (n = 3). (**A**) Ki67 and TUNEL-positive signal expression in female mice; (**B**) Ki67 and TUNEL-positive signal expression in male mice. One-way analysis of variance (ANOVA) was performed. *** *p* < 0.001, **** *p* < 0.0001 vs. blank group; ### *p* < 0.001, #### *p* < 0.0001 vs. model group. Scale bars: 50 μm.

To evaluate the effects of PA-011 on the proliferation and apoptosis of hair follicle cells, the skin sections of mice in each group were subjected to Ki67 [29] and TUNEL double immunofluorescence staining. Compared with the blank group, the expression of the ki67-positive signal in the hair follicles of mice in the male and female model groups was decreased significantly (red light), whereas the expression of the TUNEL-positive signal was increased significantly (green light), indicating that long-term DHT administration can reduce the activity oKi67 activity in hair follicles, cause apoptosis of hair follicle cells, and lead to early degeneration of hair follicles. However, positive Ki67 signal expression was increased significantly in hair follicles of PA-011 and minoxidil-treated mice, while TUNEL-positive signal expression was decreased significantly, indicating that both interventions reversed the negative effect of DHT and significantly promoted proliferation and decreased apoptosis of hair follicle cells (Figure 9).

### 3.7. Skin Transcriptomic Analysis

#### 3.7.1. Differential Gene Analysis

**Figure 10:**
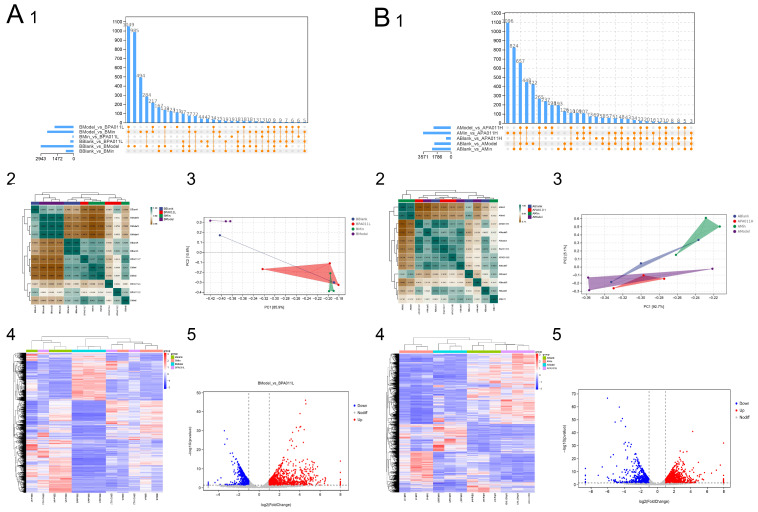
Differential gene analysis (n = 3). (**A**) Transcriptome analysis of female mice; (**B**) transcriptome analysis of male mice; (1) gene expression analysis; (2) correlation clustering heatmap; (3) PCA: principal component analysis; (4) differential gene cluster analysis; (5) differential gene volcano map.

To explore the mechanism via which PA-011 promotes hair growth in AGA model mice, we screened the RNA-seq data obtained by transcriptomic sequencing of skin tissues of male and female mice and analyzed the gene expression levels of each sample. Cluster analysis of differentially expressed genes (DEGs) (Figure 10(A1,A2,B1,B2)) showed that the correlations between the expression levels of DEGs between the model group and those of the blank group, minoxidil group, and PA-011 group were low, indicating that the model was successful and the data were reliable. Cluster processing and PCA results showed that the blank group, minoxidil group, and PA-011 group were highly correlated, whereas the correlation with the model group was low (Figure 10(A3,B3)). Differential expression gene analysis (*p* < 0.05, FC > 2) showed that 991 genes were significantly up-regulated and 1952 genes were significantly down-regulated in the female blank group compared with in the model group. Compared with the PA-011 group, 1155 genes were significantly up-regulated and 553 genes were significantly down-regulated in the model group. Compared with the model group, 927 genes were significantly up-regulated and 1191 genes were significantly down-regulated in the blank group. Compared with in the PA-011 group, 1413 genes in the model group were significantly up-regulated and 811 genes were significantly down-regulated. The numbers of common unique differential genes between the comparison groups and the numbers of common differential genes between the pair-to-pair comparison groups are shown in Figure 10(A5,B5)). (If you cannot see the content of the picture clearly, you can refer to Supplementary Material Table S1 for detailed reading).

#### 3.7.2. Protein Interaction Analysis of Differential Genes

**Figure 11:**
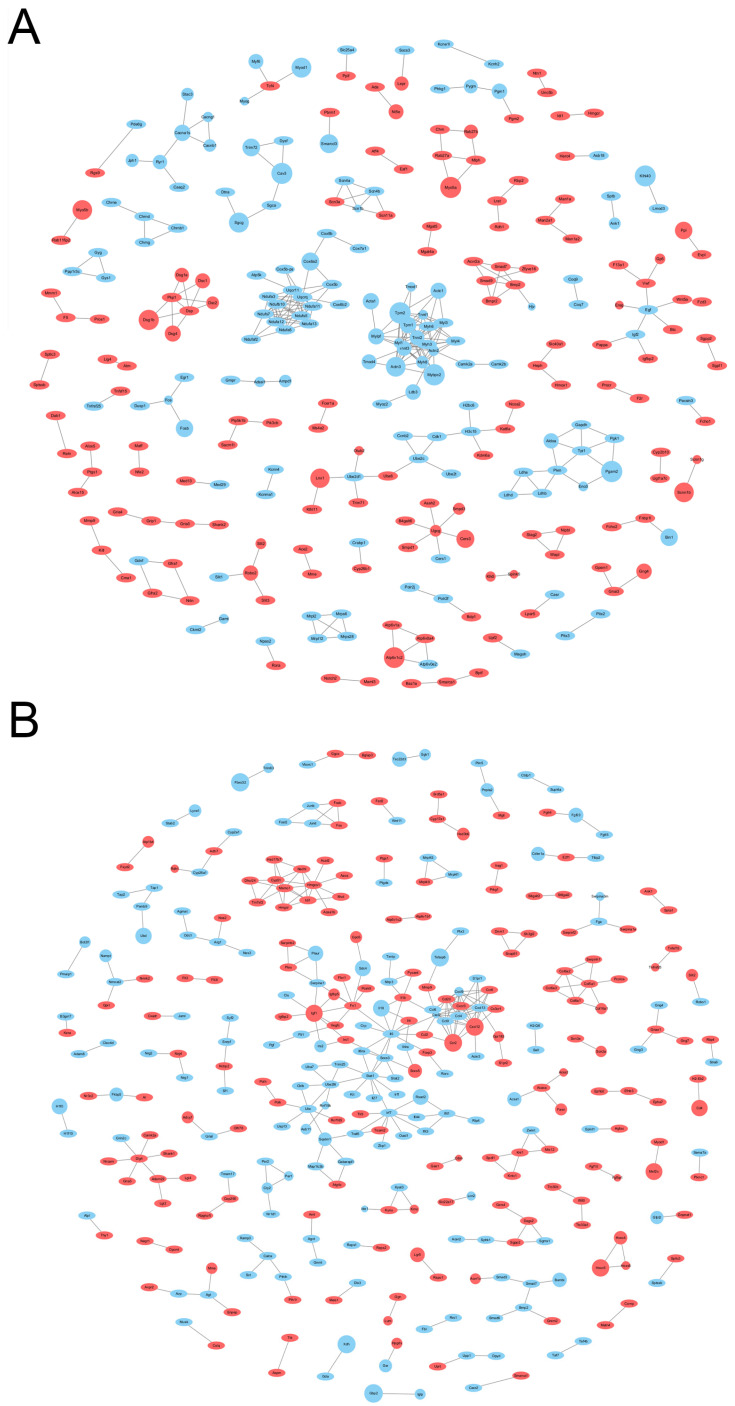
Network diagram of differential gene–protein interactions in the model group vs. Pa-011-treated group. (**A**) Female mice; (**B**) male mice. Red represents up-regulated genes, and blue represents down-regulated genes.

The differential genes of the model group and PA-011-treated group in the male and female mice were analyzed by using protein network mapping (Figure 11A,B). The PA-011 treatment effectively reversed AGA-induced gene downregulation, such as in *MAPK13*, *ll-1a*, *Il-1b*, *Il-7*, *Mmp9*, and *Mmp13*. Moreover, the treatment effectively improved Wnt11 and Vegf gene expression. (If you cannot see the content of the picture clearly, you can refer to Supplementary Material Table S1 for detailed reading).

#### 3.7.3. Transcriptome Enrichment Analysis

**Figure 12:**
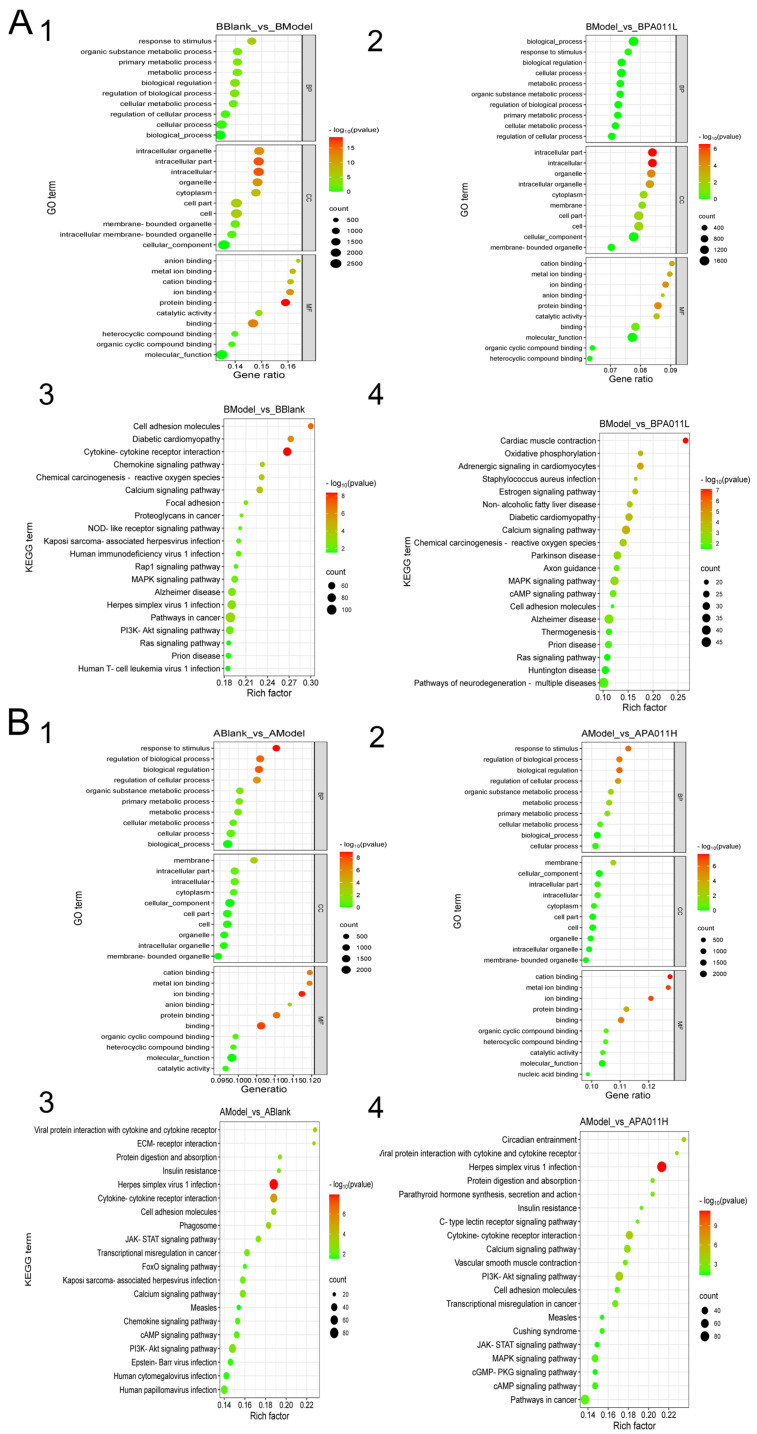
Bubble diagram of enrichment analysis. (**A**) Bubble map for enrichment analysis of female mice; (**B**) bubble map of enrichment analysis of male mice; (**A1**) GO enrichment analysis plot for BBlank vs. BModel; (**A2**) GO enrichment analysis plot for BModel vs. BPA-011L; (**A3**) KEGG enrichment analysis plot for BModel vs. BBlank; (**A4**) KEGG enrichment analysis plot for BModel vs. BPA-011L; (**B1**) GO enrichment analysis plot for ABlank vs. AModel; (**B2**) GO enrichment analysis plot for AModel vs. APA-011H; (**B3**) KEGG enrichment analysis plot for AModel vs. ABlank; (**B4**) KEGG enrichment analysis plot for AModel vs. APA-011H.

GO gene function enrichment analysis was performed in the male and female blank group vs. model group (Figure 12(A1,B1)) and between the male and female model group vs. PA-011 group (Figure 12(A2,B2)). The top 10 paths of biological process (BP), cell composition (CC), and molecular function (MF) were selected to draw a bubble map of GO function analysis. GO analysis of differential genes in the blank group vs. model group and the model group vs. PA-011 group of male and female mice showed that after PA-011 treatment, the BP results mainly showed positive regulation of biological processes, metabolic processes, response to stimuli, etc. CC results mainly showed positive correlations with cell components, organelles, and other processes. MF mainly showed positive effects on catalytic activity, protein binding, organic ring compound binding, heterocyclic compound binding, cation binding, metal ion binding, and anion binding. These differential genes were grouped into KEGG pathway groups for comparative analysis (Figure 12(A3,A4,B3,B4)), and the top 20 enriched pathways among different comparison groups were listed. The results showed that PA-011 treatment could effectively regulate several signaling pathways, such as the PI3K-Akt and MAPK signaling pathways, which play positive roles in AGA treatment. (If you cannot see the content of the picture clearly, you can refer to Supplementary Material Table S1 for detailed reading).

### 3.8. PA-011 Modulates the Wnt/β-Catenin Signaling Pathway to Promote Hair Growth in AGA Mice

**Figure 13:**
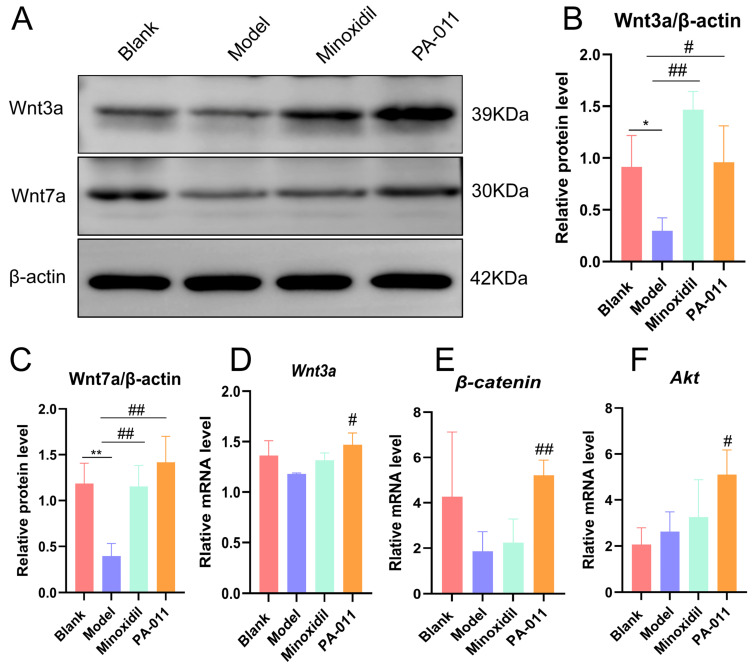
Effects of PA-011 on the expression levels of Wnt3a and Wnt7a proteins and Wnt3a mRNA in the skin of androgenetic alopecia mice (n = 3). (**A**) Protein band images; (**B**) expression level of Wnt3a protein; (**C**) expression level of Wnt7a protein; (**D**) expression level of *Wnt3a* mRNA; (**E**) expression level of *β-catenin* mRNA; (**F**) expression level of *Akt* mRNA. Data were analyzed using independent samples *t*-tests with one-way analysis of variance (ANOVA). * *p* < 0.05, ** *p* < 0.01 vs. blank group; # *p* < 0.05, ## *p* < 0.01 vs. model group. We have provided the Supplementary File with the file name Table S3.

We combined male and female subgroups for WB and RT-qPCR validation analyses. The WB results showed that the protein expression levels of Wnt3a and Wnt7a increased in the skin of the mice following PA-011 administration (Figure 13A–C). Analysis of the RT-qPCR results showed that the expression levels of *Wnt3a*, *Akt*, and *β-catenin* mRNA (Figure 13D–F) increased in the skin of mice following PA-011 administration, indicating that PA-011 promotes hair growth in AGA mice by regulating the Wnt/β-catenin signaling pathway. The results are highly similar to the transcriptomic data.

### 3.9. Skin Non-Target Metabolomics Results

#### 3.9.1. Sample Reliability Test

**Figure 14:**
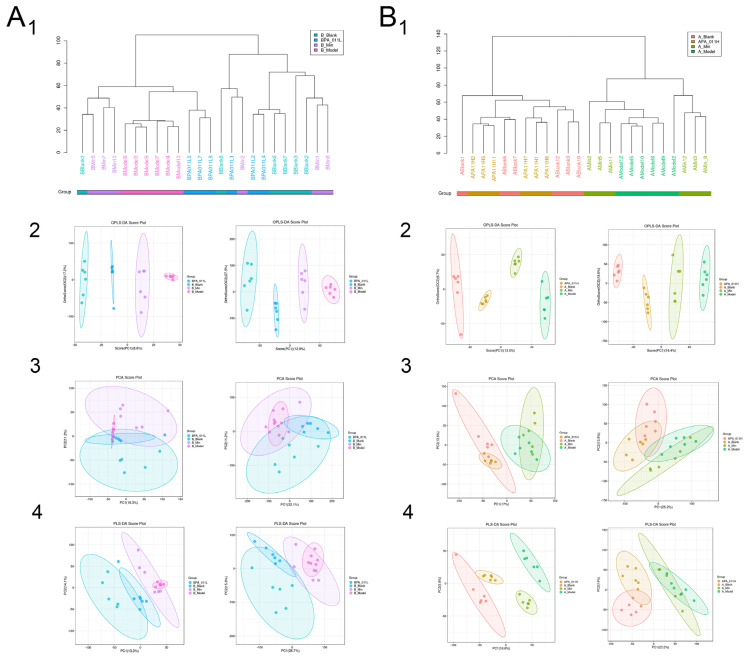
Sample reliability test (n = 6). (**A**) Female mouse samples; (**B**) male mouse sample; (1) overall sample level clustering tree; (2) POS mode and NEG mode OPLS-DA score chart; (3) PCA score chart of POS mode and NEG mode; (4) PLS-DA score chart of POS mode and NEG mode.

Based on hierarchical cluster analysis of all samples (Figure 14(A1,B1)), samples within groups had high similarity, whereas samples between groups had low similarity. OPLS-DA dimension reduction analysis, PLS-DA, and PCA were used to analyze sample data. The OPLS-DA (Figure 14(A2,B2)) and PLS-DA (Figure 14(A4,B4)) results of male and female AGA mice treated with PA-011 were significantly different from those of the AGA model group. According to the PCA results (Figure 14(A3,B3), all R2X values were >0.5, and based on R2X and R2Y, which represent the interpretation rate of the built model for X and Y matrix respectively, Q2 represents the prediction ability of the model. The closer the values to 1, the better the fitting degree of the model. Based on the results, the findings of the present study are reliable, and the model has good predictive and explanatory ability.

#### 3.9.2. Metabolite Difference Detection

**Figure 15:**
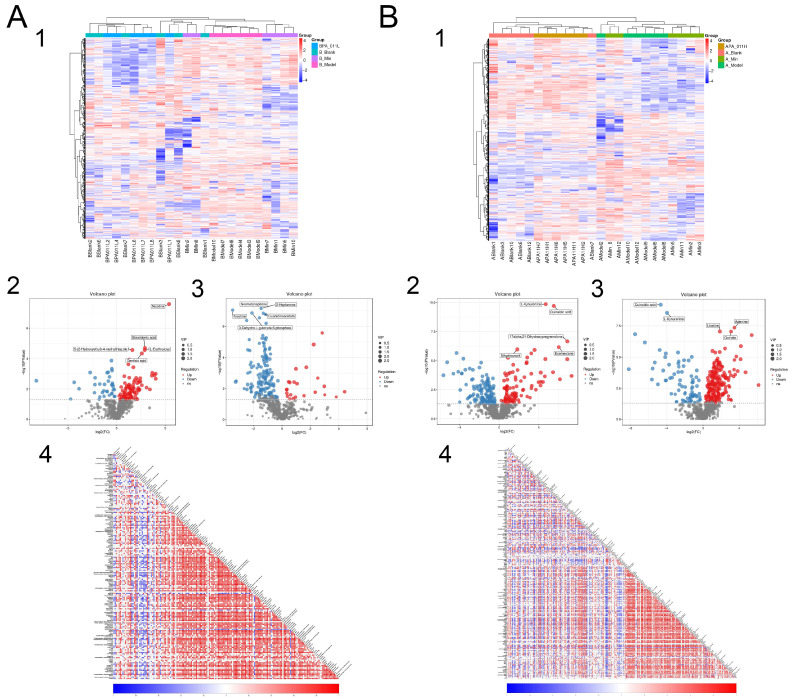
Metabolite difference detection. (**A**) Female mouse samples; (**B**) male mouse sample; (1) differential metabolite clustering heatmap; (2) volcanic map of different metabolites in blank group vs. model group; (3) volcanic map of different metabolites in PA-011 group vs. model group. (4) Correlation diagram of different metabolites in PA-011 treatment group vs. model group.

Cluster analysis of metabolites identified in all samples of male and female mice showed that PA-011-treated mice were close to mice in the blank group, with similar metabolic patterns, and they may have similar functions or participate in the same biological processes; however, they were significantly different from the model group (Figure 15(A1,B1)).

The distributions of different metabolites between the blank group and the corresponding model group of male and female mice were illustrated using a volcano map. With VIP > 1 and *p* < 0.05 as screening conditions, 132 different metabolites were found between the female blank group and its model group (Figure 15(A2)), and 176 different metabolites were found between the female PA-011-treated group and its model group (Figure 15(A3)). There were 227 different metabolites between the male blank group and its model group (Figure 15(B2)), and 292 different metabolites between the male PA-011-treated group and its model group (Figure 15(B3)). Analysis of the differential metabolites between the comparison groups showed that the differential metabolites were more similar between the blank group and the PA-011-treatment group.

Correlations among metabolites were analyzed by calculating Pearson correlation coefficients between all metabolite pinions. The results showed that correlation coefficients of the female model group and the PA-011 administration group (Figure 15(A4)) were compared with other metabolites. There were significant positive correlations for L-tryptophan with tryptamine, guanidinoacetate, ribitol, and xanthosine. In addition, corticosterone had significant negative correlations with tryptamine and pyridoxamine.

In the correlation coefficient between the male mouse model group and the PA-011 administration group (Figure 15(B4)), tetrahydrocorticosterone was positively correlated with 17alpha, 21-Dihydroxypregnenolone, 3-methyl-L-tyrosine and p-cresol. Significant negative correlations were found for inosine with kynurenic acid, tetrahydrocorticosterone, and kyotorphin; L-carnitine with ornithine, xanthosine, and kynurenic acid; and L-carnitine with tetrahydrocorticosterone and corticosterone, suggesting that the treatment of PA-011 has a positive regulation effect on the balance of metabolites. (If you cannot see the content of the picture clearly, you can refer to Supplementary Material Table S1 for detailed reading).

#### 3.9.3. Differential Metabolite Function Prediction

**Figure 16:**
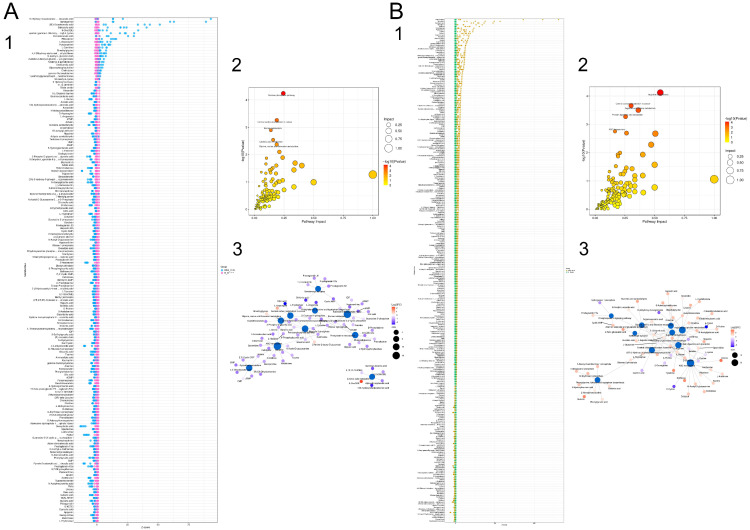
Differential metabolite function prediction. (**A**) Female mouse samples; (**B**) male mouse sample; (1) Z-score; (2) KEGG channel enrichment bubble map; (3) differential molecular number network diagram for KEGG pathways.

Z-score (standard score) was used to analyze the relative contents of metabolites, and the top six metabolites with the highest contents in the female model group were cyanuric acid, apigenin, deoxyuridine, maltotriose, L-erythrulose, and 12-KETE. The top six metabolites with the highest concentrations in the female PA-011-treated group were 16-hydroxy hexadecanoic acid, sphinganine, (9E)-octadecenoic acid, salicyluric acid, 9-oxoODE, and epsilon-(gamma-L-Glutamyl)-l-lysine (Figure 16(A1)). In addition, the top six metabolites with the highest concentrations in the male model group were 17α,21-dihydroxypregnenolone, 3-indoleacetonitrile, 3-dehydroecdysone, styrene oxide, tetrahydrocorticosterone, and (R)-10-hydroxystearate, and the top six metabolites in the male PA-011-treated group were beta-carotene, nebularine, salicyluric acid, naringenin, adenine, and maltohexaose (Figure 16(B1)).

Metabolic analysis software (v3.0.8789) was used to analyze the metabolic pathways of different metabolites in different comparison groups. A total of 141 metabolic pathways were enriched in the female model group and the female PA-011 treatment group (Figure 15(A2)), showing that the pentose phosphate pathway; central carbon metabolism; purine metabolism; linoleic acid metabolism; and glycine, serine, and threonine metabolism were significantly altered following PA-011 treatment. A total of 163 metabolic pathways were enriched in the male model group and the male PA-011 treatment group (Figure 16(B2)), which showed that after PA-011 treatment, arginine biosynthesis, metabolism of arginine and proline, protein digestion and absorption, ABC transporter, and other pathways were altered significantly.

We selected the top 20 pathways in the male and female PA-011 treatment groups and their corresponding model groups based on the number of hits and *p* < 0.05, and we established a pathway–metabolite interaction network diagram (Figure 16(A3,B3)). (If you cannot see the content of the picture clearly, you can refer to Supplementary Material Table S1 for detailed reading).

### 3.10. Combined Transcriptomic and Non-Target Metabolomics Analysis

Correlation analysis was performed on the genes and metabolites detected in each differential group. The results with Pearson correlation coefficient > 0.8 were selected for visualization, and the correlation between the total genes and metabolites expressed in each differential group was shown in a nine-quadrant diagram.

**Figure 17:**
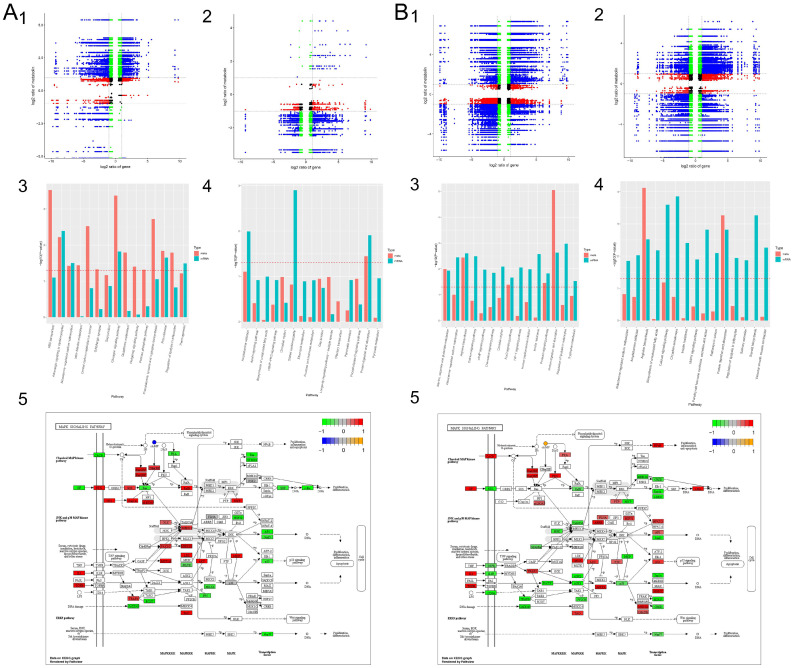
Co-analysis of transcriptomics and non-target metabolomics. (**A**) Female mice samples; (**B**) male mice samples; (1) nine-quadrant plot of differential gene and metabolite correlation analy-sis based on Pearson correlation coefficient > 0.8 for the model group vs. blank group; (2) nine-quadrant plot of differential gene and metabolite correlation analysis based on Pearson correlation coefficient > 0.8 for the model group vs. PA-011 group, Black represents that the genes and metabolites in the differential group are not differentially expressed. Red and green represent that metabolites remain unchanged while genes are up-regulated or down-regulated, or that genes remain unchanged while metabolites are up-regulated or down-regulated; (3) co-enrichment of pathways by non-target metabolomics and transcriptomic differences for the model group vs. blank group; (4) model group vs. PA-011 group non-target metabolomics and transcriptomics co-enrichment pathway; (5) model group vs. PA-011 group MAPK pathway map. The red square represents an increase, and the green square represents a decrease.

The differentially expressed metabolites and all transcripts were mapped to the KEGG pathway database simultaneously to obtain the common pathway information of the DEGs and differentially expressed metabolite mapping results. There were 165 common pathways in the female mouse model group vs. blank group (Figure 17(A1)) and 156 common pathways in the female model group compared with the PA-011 group (Figure 17(A2)). The male model group and the blank group shared 175 pathways (Figure 17(B1)), and the male model group shared 178 pathways with the PA-011 group (Figure 17(B2)). The first 15 pathways in each comparison group are shown in Figure 17(A3,A4,B3,B4). Combined transcriptomic and non-target metabolomics analysis results indicated that PA-011 may play an anti-AGA role by regulating the MAPK pathway (Figure 17(A5,B5)). (If you cannot see the content of the picture clearly, you can refer to Supplementary Material Table S1 for detailed reading).

### 3.11. PA-011 Changed the Composition of Skin Microbiota in AGA Mice

#### 3.11.1. Analysis of Skin Microbiota Diversity

We investigated the skin microbiota composition in normal, AGA-, minoxidil-, and PA-011-treated mice using 16S rRNA sequencing in males and metagenomic sequencing in females. In male mice, 70% similarity clustering yielded 1598 OTUs, encompassing 387 species, 594 genera, 276 families, 188 orders, 113 classes, and 40 phyla. Female mice clustering resulted in 11,738 OTUs, including 8292 species, 2169 genera, 727 families, 315 orders, 153 classes, and 82 phyla.

**Figure 18:**
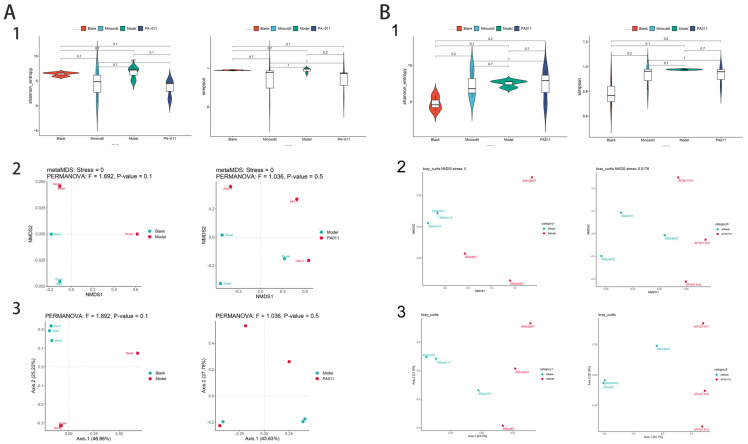
Analysis of skin microbiota diversity. (**A**) Diversity analysis of female mice; (**B**) diversity analysis of male mice; (1) Shannon and Simpson indices of the blank group vs. the model group and the model group vs. the PA-011 group; (2) NMDS analysis of the model group vs. blank group and model group vs. PA-01l group; (3) PoCA analysis of model vs. blank and model vs. PA-011.

Alpha diversity analysis showed no significant differences between groups in either male or female mice. However, Shannon and Simpson indices indicated that the PA-011 treated group’s species diversity was closer to that of the minoxidil group (Figure 18). NMDS and PCoA analyses of beta diversity revealed no significant differences between the groups, with high inter-sample separation and low intra-group separation, indicating data reliability. In addition, PA-011 treatment modified skin microbiota abundance, making it more similar to the minoxidil-positive control group.

#### 3.11.2. Genus-Level Species Analysis of Skin Microbiota

**Figure 19:**
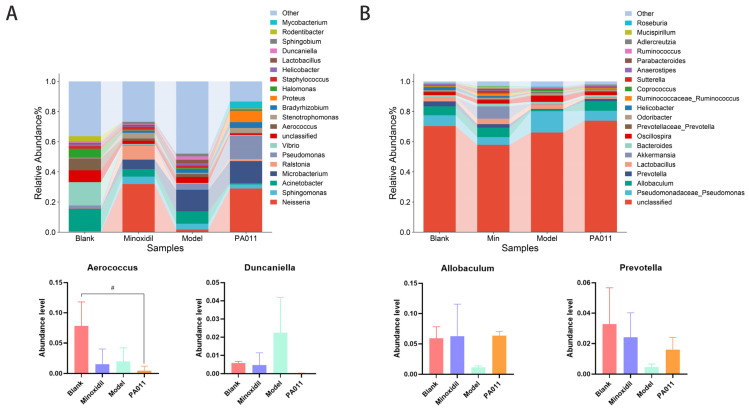
Analysis of species abundance at the genus level. (**A**) Abundance level analysis of female mice; (**B**) abundance level analysis of male mice. Data were analyzed using one-way analysis of variance (ANOVA). # *p* < 0.05 vs. blank group.

At the genus level, the skin of the model group showed a significant increase in Acinetobacter, Sphingobium, Aerococcus, and Duncaniella compared with the blank and PA-011 treated groups, which were reversed in the PA-011 group (Figure 19A). In male samples, the model group exhibited higher levels of Acinetobacter, Oscillospira, Aggregatibacter, Streptococcus, Corynebacterium, and Pseudomonadaceae_Pseudomonas than the blank and PA-011 groups. PA-011 treatment significantly increased the abundances of Allobaculum, Prevotella, and Cetobacterium (Figure 19B). We analyzed the horizontal species with the highest total abundance across groups, highlighting the species associated with AGA post-PA-011 treatment in both female and male mice (Figure 19B). In conclusion, AGA alters the skin microbiota composition, whereas PA-011 reduces pathogenic bacteria, mitigates inflammation-inducing bacteria, and effectively restructures the skin microbiota ecology.

#### 3.11.3. The Composition of Skin Microbiota Is Correlated with the Levels of HGF, VEGF, MDA, TNF-α, and IL-6 and the Activity of SOD, GSH-PX, and ALP in the Skin After AGA

**Figure 20:**
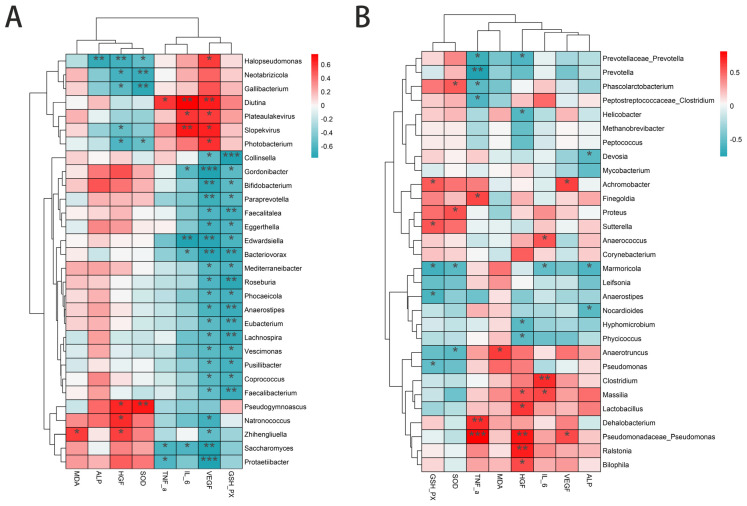
Genus-level correlation heatmap analysis. (**A**) Female mice correlation heatmap analysis; (**B**) male mice correlation heatmap analysis. Asterisks indicate significant differences in correlation: * *p* < 0.05, ** *p* < 0.01, and *** *p* < 0.001.

Given the observed changes in skin microbiota composition, we hypothesized that different species might be associated with the levels of HGF, VEGF, MDA, TNF-α, and IL-6 and activities of SOD, GSH-PX, and ALP post-AGA. ANOVA and Pearson’s correlation analyses for the skin microbiota revealed that Halopseudomonas was positively correlated with ALP in female mice. VEGF levels were significantly negatively correlated with Bacteriovorax, Protaetiibacter, Edwardsiella, and Gordonibacter, which negatively affect AGA. IL-6 and GSH-PX levels were significantly negatively correlated with probiotics such as Saccharomyces and Gordonibacter and positively correlated with pathogens such as Diutina, Plateaulakevirus, and Slopekvirus (Figure 20A).

In male mice, TNF-α levels were negatively correlated with Prevotella, Prevotellaceae_Prevotella, and Phascolarctobacterium and positively correlated with Pseudomonadaceae, Pseudomonas, Dehalobacterium, and other pathogens. IL-6 was negatively correlated with Marmoricola and positively correlated with Clostridium, Massilia, and Anaerococcus. HGF was negatively correlated with Hyphomicrobium, Phycicoccus, and Helicobacter and positively correlated with Bilophila, Lactobacillus, and Massilia (Figure 20B). These findings suggest that changes in the ecological structure of the skin microbiota after PA-011 treatment are linked to its growth-promoting effects, indicating that regulation of the skin microbiota can enhance host function and reduce inflammatory responses.

#### 3.11.4. Differences in Microbiota Composition Among Groups

To further explore the differences between the skin microbiota of AGA and normal mice, we performed an effect size (LEfSe) analysis and found statistical differences in microbial biomarkers associated with AGA through linear discriminant analysis (LDA) scores.

**Figure 21:**
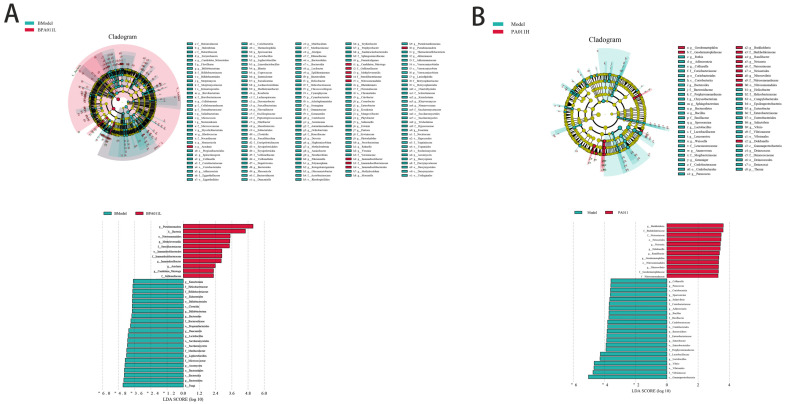
LDA analysis of skin microorganisms. (**A**) The genomic cycle map and LEfSe map of female mice and the evolutionary branching map of the model group vs. PA-011 group; (**B**) genomic and LEfSe maps of male mice and evolutionary branching maps of the model group vs. PA-011 group. The circles radiating from the inside to the outside represent the taxonomic level from phylum to the genus in the evolutionary branching diagram. Organisms with no significant differences are shown in yellow; red nodes represent microbiomes that play an important role in the red group, and green nodes represent microbiomes that play an important role in the green group. LEfSe showed significant differences in gene abundance among different groups, and histogram length showed the degree of influence of different organisms (LDA Score > 2).

The significant changes in skin microbiota between the model and PA-011 treatment groups in female mice (Figure 21A) showed that the dominant populations in the model group with the highest LDA scores were p_Bacteroidota, o_Bacteroidales, c_Bacteroidia, and p_Ascomycota. The dominant populations of the PA-011 treatment group with the highest LDA score were p_Pseudomonadota, s_Mycobacterium_tuberculosis, k_Bacteria, s_Pseudomonas_synxantha, s_Pseudomonas_sp_OST1909, etc.

Significant changes were observed in the skin microbiota of male mice in both the model and PA-011 treatment groups (Figure 21B). The dominant populations in the model group with the highest LDA scores were g_Vibrio, f_Vibrionaceae, o_Vibrionales, and c_Gammaproteobacteria. The dominant populations in the PA-011 treatment group, with the highest LDA scores, were g_Neisseria, f_Neisseriaceae, and o_Neisseriales. The above results showed that PA-011 increased the abundance of probiotics in the skin of mice, whereas it was reported that Vibrionaceae are mostly pathogenic bacteria [26], and the family members of Bacteroideaceae could produce endotoxins in the intestine [27]. Bacteroideaceae damage the gut barrier and promote inflammation by releasing toxins. These results indicate that PA-011 can ameliorate skin microbiota imbalance caused by DHT, improve the hair follicle microenvironment, and promote hair growth. (If you cannot see the content of the picture clearly, you can refer to Supplementary Material Table S1 for detailed reading).

#### 3.11.5. Prediction and Function Analysis of Microbial Composition in Each Group

**Figure 22:**
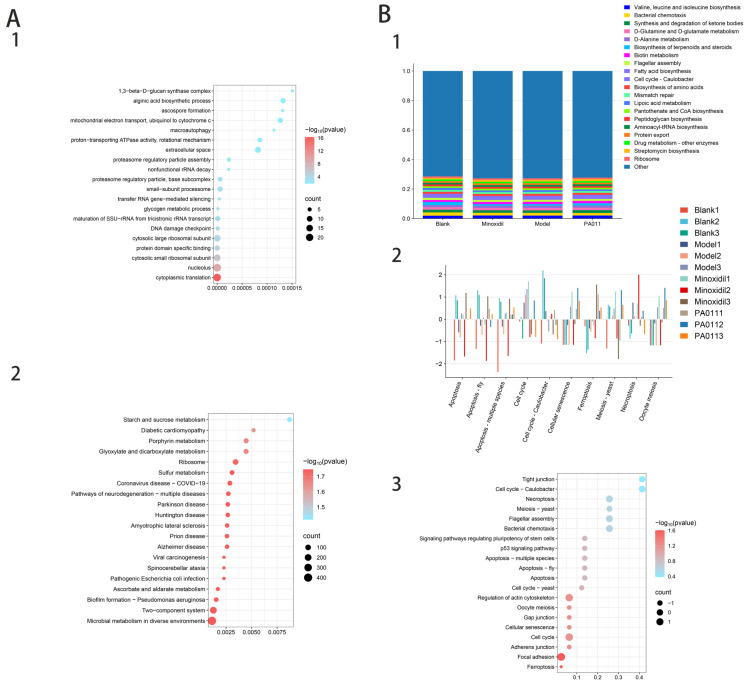
Prediction of skin microbiota function. (**A**) Prediction of skin microbiota function in female mice. (**B**) Functional prediction of skin flora in male mice. (**A1**) Bubble map of GO enrichment analysis of female skin microbiota; (**A2**) bubble map of KEGG enrichment analysis of female skin microbiota; (**B1**,**B2**) histogram of functional prediction of skin microbiota of male mice; (**B3**) bubble map of functional prediction of skin microbiota in male mice.

Functional prediction and GO enrichment analysis of female mice based on microbial genome sequences revealed that PA-011 treatment significantly affected AGA by regulating cellular protease activity, modifying cellular protein structure, and controlling glycogen metabolism (Figure 22(A1)). The functional KEGG analysis (Figure 22(A2)) indicated that PA-011 treatment effectively modulated AGA-related pathways, including starch and sucrose metabolism, ascorbic acid and dehydroascorbic acid metabolism, and glyoxylate and dicarboxylate metabolism. The MetaCyc percentage composition histogram for the male mice (Figure 22(B1)) showed key functions, such as biosynthesis of valine, leucine, and isoleucine; D-Glutamine and D-glutamate metabolism; d-alanine metabolism; biotin metabolism; fatty acid biosynthesis; and protein export, which are crucial for AGA repair. Functional annotations combined with ANOVA, Duncan’s, and Dunn’s tests were used to analyze the microbial community prediction functions in cellular processes, environmental information processing, and metabolism. The functional predictions of the Top20 samples are shown (Figure 22(B2)), with the PA-011 treatment group showing the highest expression in the pathways beneficial for AGA treatment. An integrated analysis of the pathways with significant differences between the model and PA-011 treatment groups (Figure 22(B3)) found that PA-011 treatment can regulate multifunctional cell signaling pathways by altering microbiota structure and acting on the P53 signaling pathway, effectively inhibiting apoptosis, chemotaxis, senescence, endocytosis, and oocyte meiosis in host cells. These findings suggest that PA-011 can prevent and treat AGA by modifying the ecological structure of the skin microbiota, impacting host cell apoptosis and autophagy, reducing skin inflammation, inhibiting the apoptosis of skin hair follicle cells, and promoting dermal papilla cell regeneration. (If you cannot see the content of the picture clearly, you can refer to Supplementary Material Table S1 for detailed reading).

### 3.12. Topical Application of PA-011 Demonstrated Favorable Safety Profiles

**Figure 23:**
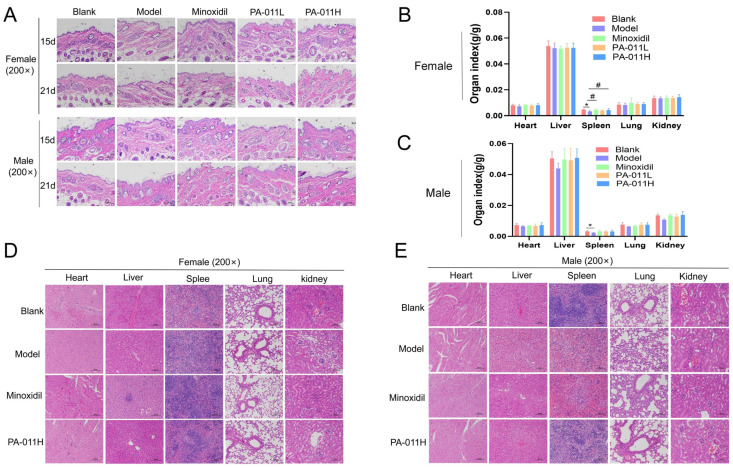
Safety evaluation of PA-011 during administration. (**A**) HE staining of skin tissue pathological sections; (**B**,**C**) organ indices of female and male mice; (**D**,**E**) HE staining of pathological sections of heart, liver, spleen, lung, and kidney in female and male mice. One-way analysis of variance (ANOVA) was performed. * *p* < 0.05 vs. blank group; # *p* < 0.05 vs. model group. Scale bars: 100 μm.

On days 15 and 21, skin samples underwent HE staining for histopathological assessment of PA-011-induced skin toxicity (Figure 23A). The blank group showed an intact stratum corneum, regular dermal collagen/muscle architecture, and normal hair follicle/sebaceous gland structures without inflammation. All experimental groups exhibited skin morphology comparable to the blank group, with no signs of toxic reactions.

Organ indices (heart, liver, lung, kidney, spleen) were evaluated in mice sacrificed on day 21. No significant differences in heart, liver, lung, or kidney indices were observed between sexes or groups (Figure 23B,C). The model group showed reduced spleen index, possibly linked to systemic stress responses. HE staining of heart, liver, spleen, lung, kidney, and testis/ovary tissues (Figure 23D,E) revealed no treatment-related toxicity in PA-011 groups compared to controls.

### 3.13. Acute Dermal Toxicity Test in Mice

**Figure 24:**
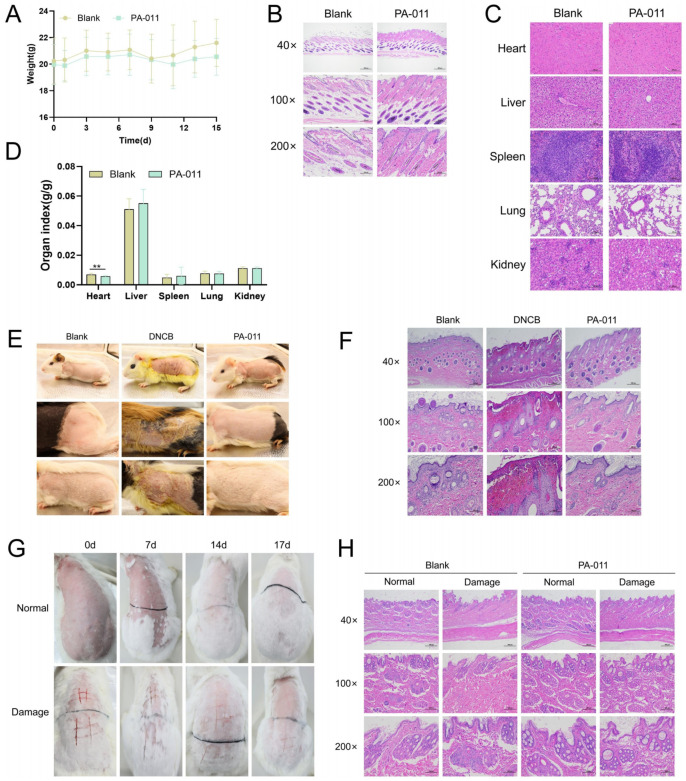
Topical application of PA-011 demonstrated favorable safety profiles. (**A**) Mouse body weight; (**B**) HE staining of skin tissue pathological sections in mice; (**C**,**D**) HE staining of pathological sections of heart, liver, spleen, lung, and kidney, and organ indices in mice; (**E**) apparent images of skin sensitization evaluation in guinea pigs; (**F**) HE staining of skin tissue pathological sections in guinea pigs; (**G**) apparent images of skin irritation evaluation in rabbits; (**H**) HE staining of skin tissue pathological sections in rabbits. Independent samples *t*-test. ** *p* < 0.05 vs. blank group. Scale bars: 500 μm, 200 μm, and 100 μm.

Results of the acute dermal toxicity test in mice showed that mice in the PA-011 group exhibited normal physical signs (Figure 24A) throughout the experiment, with no observed toxicity in organ indices or histopathological sections (Figure 24B–D).

### 3.14. Guinea Pig Skin Sensitization Test

Blank group guinea pigs displayed normal skin histology: intact epithelial layers, minimal keratinization, and no inflammatory infiltrate (Figure 24E). In contrast, the DNCB damage control group showed severe dermatitis, including stratum corneum thickening, edema, inflammatory cell infiltration (lymphocytes/eosinophils), and dermal necrosis (Figure 24F). The PA-011 group exhibited no hypersensitivity reactions, comparable to the control group.

### 3.15. Rabbit Skin Irritation Test

All rabbit groups showed normal behavior, fur condition, and respiration during the 17-day observation period. In normal skin groups, PA-011 and normal groups showed no erythema, edema, or desquamation. In wounded skin models, the PA-011 group showed faster wound healing compared to the normal group, with complete epithelialization by day 17 (Figure 24G). HE staining revealed no significant differences in corneal layer or subcutaneous tissue architecture across groups (Figure 24H).

## 4. Discussion

AGA is the most common hair loss disorder; it can start at any age after puberty, and the incidence increases with age, with up to 42% of women and 80% of men showing AGA characteristics at age ≥ 70 [30]. The availability of new drugs for treating AGA has not increased significantly, and the high cost of treatments and their limited efficacy do not meet the needs and expectations of the majority of patients [31]. Furthermore, minoxidil and finasteride, the two most commonly used drugs for treatment of AGA, have been reported to have relatively apparent side effects [32,33,34]. Therefore, research and development for new, effective, and safe AGA treatment drugs has important practical significance.

Studies have shown that the occurrence and development of AGA are related to various factors [35], among which, an increase in DHT is considered the main cause. Androgens have been shown to enhance the pro-apoptotic signaling pathway, which can inhibit the development of hair follicles or lead to the apoptosis of follicular keratinocytes by reducing typical Wnt signaling activity [36]. Apoptosis-related proteins inhibit the proliferation of dermal papilla cells, leading to premature hair follicle maturation and further hair loss [37]. AGA is characterized by damaged hair follicle progenitor cells, but with a relatively intact pool of HFSCs, which allows AGA to be treated [38].

In the present study, an AGA model was established by subcutaneous injection of DHT into mice [39]; in addition, PA-011, an *P. americana* extract, was applied to the skin of the shedding area of mice. Both PA-011- and minoxidil-treated mice had darker skin in the depilating area sooner than in the model group. When the hair cycle of C57BL/6J mice transitions from resting period to growth period, skin pigmentation will occur due to melanin production in hair follicle cells (HFs) [40]. This indicates that PA-011- and minoxidil-treated mice entered the growth phase earlier and had a faster hair growth rate than model mice. Skin color change scores, hair coverage scores, hair length measurements, and hair weighing results all showed that PA-011 promoted hair growth significantly in AGA mice.

HE staining results showed that PA-011 could directly affect hair follicle development in skin tissue. The number of hair follicles in PA-011-treated mice was significantly higher than that in the model group, and a large number of high-density enlarged hair bulbs could be seen in the subcutaneous tissue. The results showed that PA-011 intake improved the hair follicle miniaturization caused by DHT, stimulated the hair growth cycle, and increased the proportion of hair follicles in the growth phase. In the results of Ki67 and TUNEL immunofluorescence staining, the positive expression rate of Ki67 in the skin tissue of mice treated with PA-011 was significantly higher than that of the model group, whereas the positive expression rate of TUNEL was significantly lower, indicating that PA-011 promoted the proliferation and reduced the apoptosis of hair growth-related cells in hair follicles. In addition, studies have shown that the induction of the hair growth phase is associated with rapid and abundant cell proliferation [41]. This suggests that PA-011 promotes hair follicle activation and hair follicle cell proliferation, thus contributing to the transition of HFs in the resting phase to the growing phase.

Studies have shown that oxidative stress can lead to hair loss through apoptosis of hair follicle cells [42], and peroxide damage is a potential factor involved in microvascular dysfunction; furthermore, blood vessel formation can accelerate the induction of hair growth period and increase hair follicles and hair shaft diameter [43]. In the experimental results, compared with the model group, the MDA level in the skin tissue of mice treated with PA-011 was decreased significantly, whereas the SOD level was increased significantly, and GSH-PX enzyme activity was also increased. The results indicated that PA-011 can alleviate oxidative stress through antioxidant activity, in turn inhibiting production or eliminating excess reactive oxygen species, effectively reshaping the oxidative microenvironment around follicles in the hair shedding area.

PA extract has been reported to have anti-inflammatory effects in various inflammation-related diseases [8,44,45], which can increase the expression level of VEGF and promote wound repair. VEGF can induce the proliferation and migration of DPCs or the formation of perifollicular blood vessels and regulate the hair cycle, and high expression of VEGF can reduce DHT-induced apoptosis significantly [46]. In hair follicle diseases, such as AGA and alopecia areata, VEGF expression in the scalp of patients is significantly reduced or even disappears. HGF and transforming growth factor are also involved in AGA hair repair [47]. The Wnt/β-catenin signaling pathway can regulate the formation of hair follicles and the function of the dermal papilla, stimulate the proliferation and differentiation of hair follicle stem cells, and activate the Wnt/β-catenin signaling pathway, which is conducive to improving the atrophy and number reduction caused by AGA [48]. In the present study, both PA-011 and minoxidil decreased the expression levels of TNF-α and IL-6 significantly in AGA mouse skin and effectively increased the expression levels of VEGF and HGF proteins. Additionally, RT-qPCR and Western blot analyses showed that PA-011 upregulated the mRNA expression levels of Akt, Wnt3ah, and β-catenin and increased the protein expression levels of Wnt3a and Wnt7a. The results suggest that PA-011 activates the Wnt/β-catenin signaling pathway while exerting anti-inflammatory effects and remodeling the microvascular system around the follicles.

Because PA-011 is a mixture of many substances, studying its molecular mechanism is challenging. In the present study, LC-MS/MS and polypeptidyomics were used to explore its components, and the main active components and molecular mechanisms of PA-011 anti-AGA were investigated using network pharmacological prediction combined with experimental verification of the identified components. The network pharmacology results showed that 20 small molecule compounds, including sotalol, 17a-estradiol, dehydroepiandrosterone, and 10 peptides, may be the key components of PA-011 that can be used in AGA therapy. In the network pharmacological results, the differential genes were enriched mainly in the PI3K-Akt and MAPK signaling pathways, which is highly consistent with the transcriptomic results. Li et al. [49] showed that activation of PI3K-Akt and MAPK signaling pathways could stimulate DPC and DSC proliferation and promote cell cycle transition from the G1 phase to S phase. Ji et al. [50] reported that the PI3K-Akt signaling pathway plays a crucial role in the regeneration process of hair follicles, which provides further theoretical support for PA-011 effectiveness against AGA.

Non-targeted metabolomics analysis results showed that PA-011 exerts anti-AGA effects mainly by regulating the pentose phosphate pathway, arginine biosynthesis and metabolism, cysteine and methionine metabolism (antioxidant) [51], and tryptophan biosynthesis pathway. The pentose phosphate pathway is the main source of nicotinamide adenine dinucleotide phosphate oxidase, which can clear ROS in fatty acid synthesis [52], in turn inhibiting oxidative stress [53]. Clinical studies have shown that the levels of essential amino acids in the blood of AGA patients are low, suggesting that hair loss may be related to the production or acquisition of amino acids. The main pathways obtained by the combined analysis of transcriptomics and metabolomics are the MAPK and Hedgehog signaling pathways. The Hedgehog signaling pathway can reprogram fibroblasts in the hair follicle niche to a hyper-activated state [54], thus promoting hair regeneration. Consequently, PA-011 could regulate oxidative stress and inflammation by regulating the pentose phosphate pathway, amino acid synthesis, and metabolism, which then regulate cell proliferation and apoptosis, thereby improving AGA.

The skin has a complex microbiota ecosystem, and human skin is inhabited by unique microbial communities [55]. Studies have shown that in the scalp microbiota of patients with alopecia areata, Bacillus acnes is significantly increased, whereas Staphylococcus epidermidis is significantly decreased [56], leading to the aggravation of alopecia areata. When Lactobacillus *Lacticaseibacillus rhamnosus* overgrows in the skin [57], the ecological balance between Staphylococcus and Corynebacterium is disrupted, and an increase in the abundance of cyanobacteria causes hair loss and aggravates the development of AGA [58]. Therefore, maintaining the ecological balance of microbiota in the skin and hair is important for promoting hair growth and preventing AGA.

The results showed that the skin microbiota mediates the inflammatory response and two important processes of epidermal development and differentiation. Elevated levels of inflammatory factors detected in the skin of AGA mice may also be a cause of HF miniaturization. In the model group, the abundances of Bacillus and Staphylococcus increased; those of Corynebacterium, Actinomyces, and *Euryarchaea* decreased; and cyanobacteria showed excessive growth. Treatment with PA-011 effectively increased the abundance of the probiotic bacteria *Prevotella*, *Prevotellaceae_prevotella*, and *Phascolarctobacterium*, which were highly correlated with inflammation. The abundance of pathogenic bacteria, such as *Diutina*, *Plateaulakevirus*, and *Slopekvirus*, decreased, and the overgrowth of Bacteroides slowed.

Taken together, these results indicate that PA-011 can effectively promote the proliferation of HF cells, shorten the transition time of HFs from the resting to anagen phase, and significantly promote hair growth in AGA mice. The mechanism of action of PA-011 is to activate the Wnt/β-catenin signaling pathway; regulate the P13-Akt and MAPK signaling pathways; inhibit harmful bacteria with pro-inflammatory effects in the skin; increase the abundance of probiotic flora; downregulate or inhibit inflammatory factors in the skin; and increase the expression of HGF, ALP, Ki67, and VEGF in the skin, promoting hair growth. Furthermore, topical application of PA-011 demonstrated favorable safety profiles. Multi-dimensional evaluations including acute toxicity tests, skin sensitization tests, and skin irritation tests showed that PA-011 did not induce significant systemic toxicity, immune hypersensitivity, or local skin damage at the tested doses, providing a safety basis for its further clinical application. To enhance transdermal drug absorption efficiency and bioavailability, subsequent studies are proposed to formulate PA-011 into hydrogel systems with both injectable compliance and self-healing functionality [59] or construct nanogel delivery systems with superior biosafety [60], thereby advancing the clinical translation of this drug.

## 5. Conclusions

Integrating network pharmacology, in vivo experiments, multi-omics analyses, and safety evaluations, this study demonstrates that PA-011 extract effectively promotes hair growth in AGA models with favorable safety profiles. Mechanistically, PA-011 modulates skin microbiota composition via Wnt/β-catenin, PI3K-Akt, and MAPK pathways; reduces cutaneous inflammation and oxidative stress; and stimulates HF cell proliferation. These effects drive HF transition from the telogen to anagen phases, culminating in robust hair regeneration. PA-011 exhibits no significant systemic toxicity or allergic reactions, establishing its potential as a safe and efficacious therapeutic for AGA.

## Figures and Tables

**Table 1:** PA-011 gradient elution procedure for small and medium molecular compounds.

Mode	Time (min)	Mobile Phases
Pos	0~1	2% B2
	1~9	2~50% B2
	9~12	50~98% B2
	12~13.5	98% B2
	13.5~14	98~2% B2
	14~20	2% B2
Neg	0~1	2% B3
	1~9	2~50% B3
	9~12	50~98% B3
	12~13.5	98% B3
	13.5~14	98~2% B3
	14~17	2% B3

**Table 2:** Gradient elution procedure for polypeptide components in PA-011.

Time (min)	Mobile Phases
0~7	8% B
7~55	12% B
55~56	30% B
65~66	40% B
66~80	95% B
80	95% B

**Table 3:** Primer sequences.

Name		Primer Sequence (5′-3′)
*Gapdh*	Forward	CCTCGTCCCGTAGACAAAATG
	Reverse	TGAGGTCAATGAAGGGGTCGT
*β-catenin*	Forward	GGACCCCAAGCCTTAGTAAACA
	Reverse	TTATATCATCGGAACCCAGAAGC
*Wnt3a*	Forward	ATCTGGTGGTCCTTGGCTGTG
	Reverse	ACTCCTGGATGCCCGCTTT
*Akt*	Forward	CTTCCTCCTCAAGAACGATGGC
	Reverse	TGTCTTCATCAGCTGGCATTGT

**Table 4:** The top 20 most correlated small molecule compounds.

Number	Name	ID	Molecular Formula	Structures
1	(R)-Methysticin	PA3	C_15_H_14_O_5_	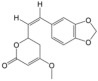
2	(S)-N-Methylcoclaurine	PA5	C_18_H_21_NO_3_	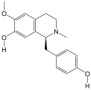
3	3-Hydroxyflavone	PA23	C_15_H_10_O_3_	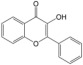
4	3-Methylindole	PA28	C_9_H_9_N	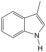
5	4-Methylcatechol	PA38	C_7_H_8_O_2_	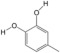
6	5,7-Dihydroxyflavone	PA39	C_15_H_10_O_4_	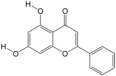
7	6-Hydroxymelatonin	PA42	C_13_H_16_N_2_O_3_	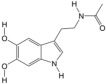
8	13S-hydroxyoctadecadienoic acid	PA48	C_18_H_32_O_3_	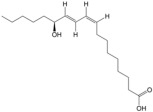
9	17a-Estradiol	PA49	C_18_H_2_4O_2_	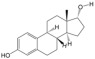
10	Acetylcholine	PA50	C_7_H_16_NO_2_	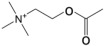
11	all-trans-Retinoic acid	PA51	C_20_H_28_O_2_	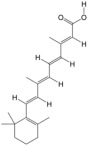
12	Alpha-Linolenic acid	PA52	C_18_H_30_O_2_	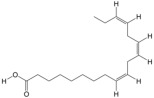
13	Caffeate	PA59	C_9_H_8_O_4_	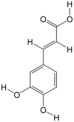
14	Dehydroepiandrosterone	PA66	C_19_H_28_O_2_	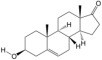
15	Dodecanedioic acid	PA72	C_12_H_22_O_4_	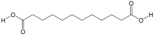
16	Misoprostol	PA106	C_22_H_38_O_5_	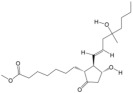
17	Pipecolic acid	PA131	C_6_H_11_NO_2_	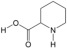
18	Propylparaben	PA132	C_10_H_12_O_3_	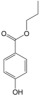
19	Sotalol	PA142	C_12_H_20_N_2_O_3_S	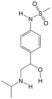
20	Stearidonic acid	PA143	C_18_H_28_O_2_	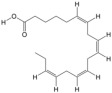

**Table 5:** The top 10 polypeptide sequences with degree value.

Number	Peptide	ID	Charge
1	GGGAGGGAGGFGGGAGGGYR	peptide18	1
2	AAKAAHFAAYGAA	peptide24	1.5
3	FYGVVRAP	peptide70	1
4	FGGANR	peptide81	1
5	YAPR	peptide86	2
6	FGGGGAGGFGGGAGGR	peptide97	1
7	PFFPGLVK	peptide100	1
8	YPYAPR	peptide109	2
9	AAKAAHF	peptide125	1.5
10	GYHVR	peptide126	1.5

## Data Availability

All the data are uploaded to the Mendeley Data database; the data set name is the same as the article title, DOI: 10.17632/jnn2vj6yb8.3.

## Supplementary Materials

The following supporting information can be downloaded at: https://www.mdpi.com/article/10.3390/biology14070831/s1, Table S1: Figure details; Table S2: PA-011 LC-MS identification sheet; Table S3: Western blotting Original image; Table S4: Quantitative information on 20 important compounds screened from PA-011; Table S5: De novo peptides in PA-011; Table S6: Peptides in PA-011.

## Abbreviations

The following abbreviations are used in this manuscript:

AGA	Androgenetic alopecia
ALP/AKP	Alkaline phosphatase
AR	Androgen receptor
BP	Biological process
CC	Cell composition
DHT	Dihydrotestosterone
DPCs	Ermal papilla cells
E2	Estradiol
GO	Gene Ontology
GSH-PX	Glutathione peroxidase
HE	Hematoxylin and Eosin
HFs	Hair follicle cells
HGF	Hepatocyte Growth Factor
IL-1α	Interleukin-1α
IL-6	Interleukin 6
KEGG	Kyoto Encyclopedia of Genes and Genomes
LC-MS/MS	Liquid Chromatography Tandem Mass Spectrometry
MDA	Malondialdehyde
MF	Molecular function
OPLS-DA	Orthogonal partial least squares discriminant analysis
PA	*Periplaneta americana*
PAE	*P. americana* extract
PCA	Principal component analysis
PLS-DA	Partial least squares discriminant analysis
PPI	Protein interaction
SEM	Standard error of the mean
SOD	Superoxide dismutase
TGF-β 1	Transforming growth factor-β1 protein
TNF-α	Tumor necrosis factor-α
VEGF	Vascular endothelial growth factor
DNCB	Dinitrochlorobenzene
