# Supplementary material for: Exploring the Efficacy and Potential Mechanisms of Topical Periplaneta americana (L.) Extract in Treating Androgenetic Alopecia in a Mouse Model: A Systems Pharmacology and Skin Microbiome Analysis
Source: Biology (Basel). 2025 Jul 8;14(7):831. doi: 10.3390/biology14070831 (PMC12292697; doi:10.3390/biology14070831)
Supplement: Supplementary file 1 [file biology-14-00831-s001.zip › Table S3 Western blotting Original image.pdf]

proteins	strip chart	grayscale value
Wnt3A	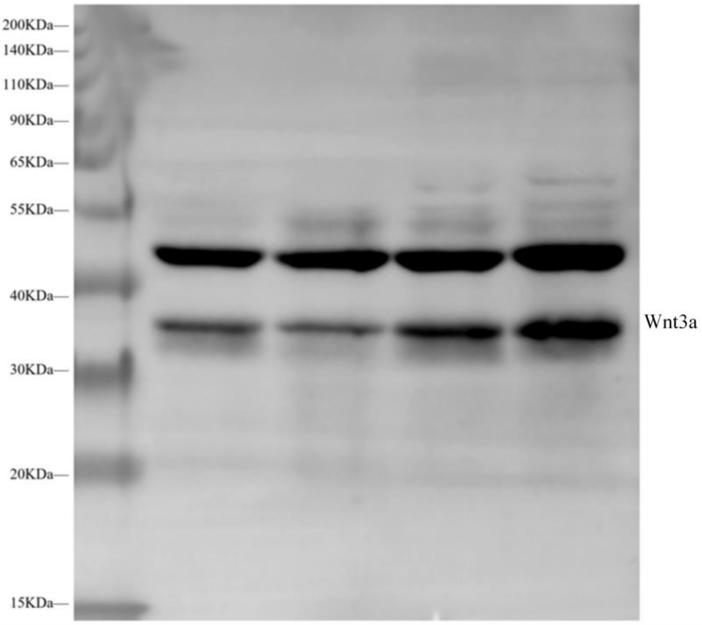	Blank:229363; Model:171643; Minoxidil:393123; PA-011:513920
Wnt7a	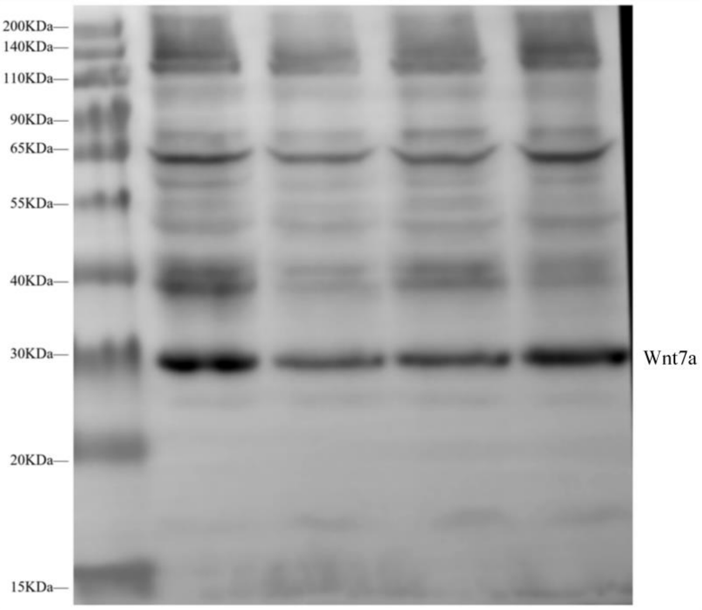	Blank:372164; Model:177742; Minoxidil:203026; PA-011:258664

β-actin	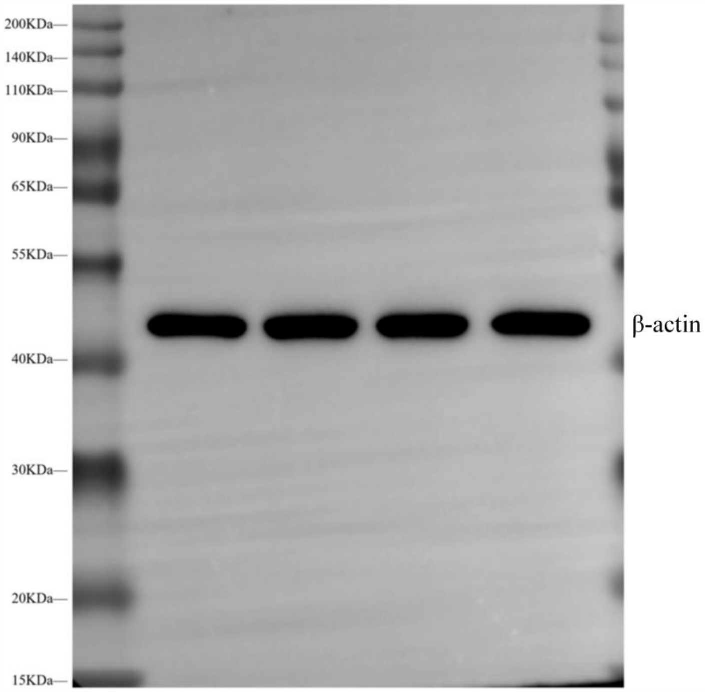	Blank:481402;<br>Model:480650;<br>Minoxidil:481925;<br>PA-011:497978
Notes	The first point (blank group), the second point (model group), the third one (minoxidil group), and the third point (PA-011 group)	

proteins	strip chart
Wnt7a	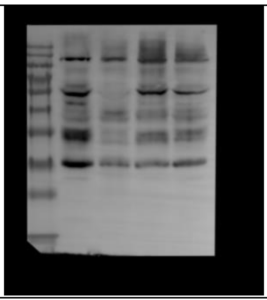
	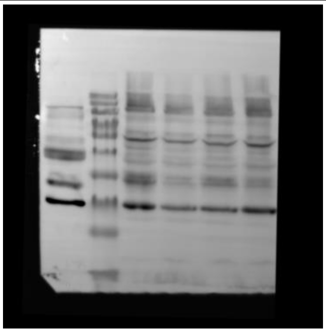

	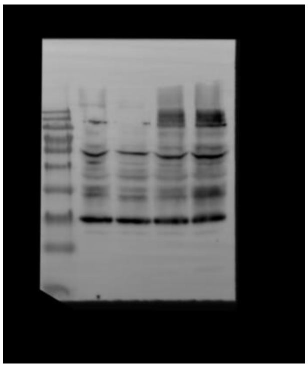
Wnt3a	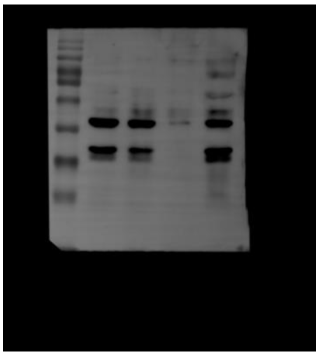
	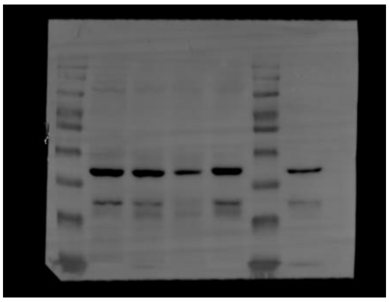
	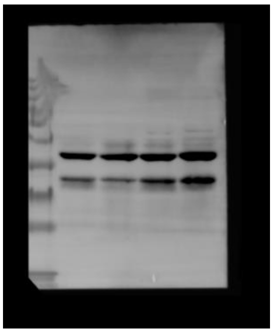
β-actin	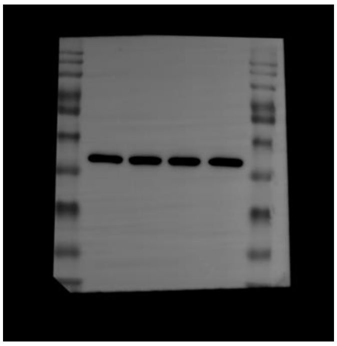

	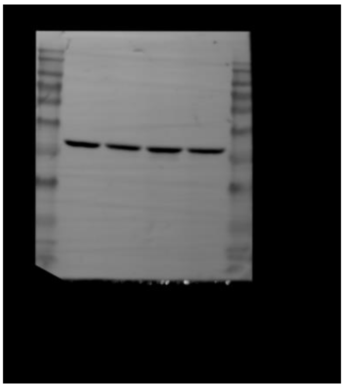
	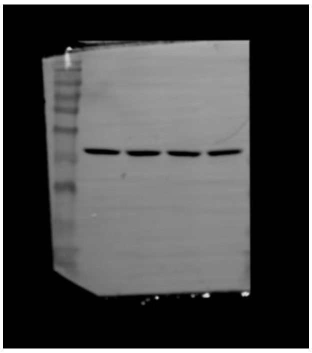
Note	The first point (blank group), the second point (model group), the third one (minoxidil group), and the third point (PA-011 group)
